# TMEM106B C‐terminal fragments aggregate and drive neurodegenerative proteinopathy in transgenic Caenorhabditis *elegans*

**DOI:** 10.1002/alz.14468

**Published:** 2024-12-23

**Authors:** Ruben Riordan, Aleen Saxton, Marina Han, Pamela J. McMillan, Rebecca L. Kow, Nicole F. Liachko, Brian C. Kraemer

**Affiliations:** ^1^ Geriatrics Research Education and Clinical Center Veterans Affairs Puget Sound Health Care System Seattle Washington USA; ^2^ Division of Gerontology and Geriatric Medicine Department of Medicine University of Washington Seattle Washington USA; ^3^ Graduate Program in Neuroscience University of Washington Seattle Washington USA; ^4^ Department of Psychiatry and Behavioral Sciences University of Washington Seattle Washington USA; ^5^ Department of Laboratory Medicine and Pathology University of Washington Seattle Washington USA

**Keywords:** aging, dementia, frontotemporal lobar degeneration, neurodegeneration, progranulin, proteinopathy, tau, TMEM106B

## Abstract

**INTRODUCTION:**

Genetic variation in the lysosomal and transmembrane protein 106B (TMEM106B) modifies risk for several neurodegenerative disorders, especially frontotemporal lobar degeneration (FTLD). The C‐terminal (CT) domain of TMEM106B occurs as fibrillar protein deposits in the brains of dementia patients.

**METHODS:**

To determine the TMEM CT aggregation propensity and neurodegenerative potential, we generated transgenic *Caenorhabditis elegans* expressing the human TMEM CT fragment aggregating in FTLD cases.

**RESULTS:**

Pan‐neuronal expression of human TMEM CT in *C. elegans* causes severe neuronal dysfunction driving neurodegeneration.  Cytosolic aggregation of TMEM CT proteins accompanied by behavioral dysfunction and neurodegeneration. Loss of *pgrn‐1* did not modify TMEM CT phenotypes suggesting TMEM CT aggregation occurs downstream of PGRN loss of function. The mechanistic drivers of TMEM106B proteinopathy appear distinct from known modifiers of tauopathy.

**DISCUSSION:**

Our data demonstrate that TMEM CT aggregation can kill neurons. TMEM106B transgenic *C.elegans* provide a useful model for characterizing TMEM106B proteinopathy‐mediated neurodegeneration in FTLD.

**Highlights:**

- Pan‐neuronal expression of human TMEM106B C‐terminal fragments (TMEM CT) in *C. elegans* neurons drives a suite of disease‐related phenotypes useful for modeling the molecular and cellular features of TMEM106B neuropathology.
- TMEM CT expression results in extensive TMEM aggregation and accumulation of highly detergent insoluble protein species.
- TMEM CT expression causes moderate to severe neuronal dysfunction dependent on TMEM CT abundance as measured by stereotypical behavioral readouts.
- TMEM CT expression drives significant neurodegenerative changes.
- Dendra2 tagged TMEM exhibits similar properties to untagged TMEM allowing ready visualization of the protein.
- TMEM CT aggregates accumulate adjacent to but not within lysosomes.
- PGRN loss of function does not impact TMEM CT toxicity.
- Modifiers of tau and TDP‐43 proteinopathies have little impact on TMEM CT‐related neurodegenerative phenotypes.

## 1 BACKGROUND

### 1.1 Impact of TMEM106B C‐terminal fragment aggregation on neurodegenerative disease

Diseases exhibiting accumulation of specific misfolded and aggregated proteins are generically referred to as proteinopathies and occur in association with neuronal degeneration and aging.^1^, ^2^, ^3^, ^4^ Protein aggregation of tau, amyloid beta peptide, α‐synuclein, and TAR DNA binding protein 43 (TDP‐43) represent well‐characterized drivers of diverse proteinopathy disorders associated with dementia that include Alzheimer's disease (AD), dementia with Lewy bodies (DLB), and frontotemporal lobar degeneration (FTLD).^5^, ^6^, ^7^ Accruing evidence suggests neurodegenerative events often involve co‐occurring proteinopathies.^8^, ^9^ However, the molecular relationships between co‐occurring pathological proteins remain incompletely understood. Improving interventions for proteinopathy disorders requires a better understanding of the interplay between distinct pathological protein species.^10^, ^11^ The transmembrane protein 106B (TMEM106B) has recently emerged as a new pathological protein implicated in multi‐proteinopathies across a range of neurodegenerative conditions and in advanced aging.^12^, ^13^, ^14^, ^15^, ^16^

TMEM106B is a single pass, lysosomal, type II transmembrane protein highly expressed in neurons and glial cells of the central nervous system (CNS) that plays an important role in regulating lysosomal size, mobility, and acidity.^17^, ^18^, ^19^, ^20^ Decreased and increased expression of TMEM106B has been associated with severe lysosomal defects in neurons and oligodendrocytes.^17^, ^21^, ^22^, ^23^ TMEM106B was initially identified as a genetic risk factor for FTLD with TDP‐43 (FTLD‐TDP), with greater genetic impact on carriers of PGRN mutations.^18^, ^24^, ^25^, ^26^, ^27^, ^28^ Single nucleotide polymorphisms (SNPs) in TMEM106B have since been identified as risk modifiers for a variety of neurodegenerative diseases including AD, hippocampal sclerosis (HS), and Parkinson's disease (PD).^18^, ^24^, ^29^, ^30^, ^31^, ^32^

With the understanding that lysosomal dysfunction occurs during neurodegeneration,^33^, ^34^ determining the mechanistic role of lysosomal pathways in neuroprotection against proteinopathy remains unclear. Specifically, the molecular role TMEM106B plays in neurodegenerative disease requires further investigation. Several studies have explored the link between TMEM106B expression and neurodegenerative conditions, often with contradicting results. An analysis of TMEM106B SNPs in FTLD‐TDP indicated that risk‐associated alleles increase protein levels through transcriptional activation.^23^ Further, overexpression of TMEM106B in neurons was shown to increase lysosomal dysfunction caused by GRN deficiency.^35^ However, studies assessing the potential for TMEM106B reduction to ameliorate effects of PGRN loss in FTLD instead showed exacerbations in lysosomal dysfunction and FTLD‐related symptoms.^21^, ^36^ Different studies of TMEM106B risk alleles in AD cases reported both an increase and a decrease in TMEM106B expression in brain tissue.^26^, ^29^

Recently, due to advances in cryogenic electron microscopy (cryo‐EM) previously unidentified, cytoplasmic protein aggregates in the brains of a diverse range of neurodegenerative diseases including FTLD, DLB, progressive supranuclear palsy (PSP), and PD were discovered to contain homotypic fibrils of the CT fragment of TMEM106B localized to the cytosol.^12^, ^13^, ^14^, ^15^, ^16^ These TMEM106 fibrils co‐occur with other neuropathological fibrils of tau, α‐synuclein, or TDP‐43. It is predicted that this CT domain of TMEM106B is generally cleaved and degraded through normal lysosomal function.^37^ Conditions of increased lysosomal burden may lead to accumulation and aggregation of this CT protein in the cytoplasm, contributing to neurotoxicity in disease. In this study, we aim to model cytoplasmic TMEM CT aggregation in vivo, and assess the potential for this aggregation to impact neuronal degeneration in human disease.

Herein, we describe the first live organism transgenic model of TMEM106B CT proteinopathy generated by expressing the TMEM106B CT aggregation‐prone fragment constituting the fibrillar core in FTLD cases (TMEM CT). As with other aggregating proteins including amyloid beta, tau, TDP‐43, and alpha‐synuclein, TMEM CT causes obvious neuronal dysfunction in the form of uncoordinated locomotion. We show that TMEM106B fragments drive a suite of neurodegenerative phenotypes in *Caenorhabditis elegans*, representing the first evidence of the neurodegenerative nature of TMEM106B proteinopathy.

## 2 METHODS

### 2.1 *C. elegans* strains and transgenics

*C. elegans* transgenic strains and information are listed in Table S1.

### 2.2 Plasmids

To generate transgenes expressing the CT fragment of human TMEM106B driven by a pan‐neuronal *snb‐1* promoter, a *C. elegans* codon‐optimized DNA sequence encoding the CT fibrillar core fragment of wild‐type TMEM106B (amino acids 120‐254 of NP_001127704) was purchased from Integrated DNA Technologies (IDT). The parental *snb‐1p *vector was previously constructed^38^ by inserting the *snb‐1* promoter sequences into the *Hin*dIII and *Bam*HI sites of MCS I of plasmid pPD49.26 (a generous gift from Dr. A. Fire, Stanford University, Palo Alto, CA).

### 2.3 Transgenics and strains

N2 (Bristol) was used as wild‐type *C. elegans* and maintained as previously described.^39^ The marker transgene *myo‐3p*::mCherry was used at a concentration of 20 ng/µL as a co‐injection marker as previously described.^38^ The *snb‐1p*::hTMEM106B‐core and *snb‐1p*::dendra2hTMEM106B‐core transgenes described above were microinjected into N2 at a concentration of 150 ng/µL and 75 ng/µL, respectively, as previously described^40^ to produce animals expressing TMEM106B and mCherry transgenes as extrachromosomal arrays. Extrachromosomal transgenes were integrated by exposing animals to ultraviolet (UV) radiation in a Stratalinker for 0.8 min. The progeny of irradiated animals was screened for 100% transmission of the transgene and integrated strains were backcrossed to N2 at least three times.

**RESEARCH IN CONTEXT**

1. **Systematic review**: Genetic variation in the TMEM106B gene drives risk for many aging‐related dementia disorders. C‐terminal fragments of TMEM106B (TMEM CT) protein form fibrillar aggregates in the brains of dementia patients, but the relative toxicity of the accumulating TMEM106B proteins remains uncertain. No animal models exist in the published literature that exhibit neuronal TMEM CT aggregation.
2. **Interpretation**: Here we began investigating the protein aggregation propensity of TMEM106B by generating a new transgenic model of TMEM106B proteinopathy. This model has allowed us to assess the neurodegenerative potential of aging‐dependent TMEM106B fibrillization. Our work clearly demonstrates that TMEM CT aggregation kills neurons. TMEM CT aggerates accumulate adjacent to, but outside of lysosomes. TMEM CT aggregation appears to be downstream of PGRN loss of function.
3. **Future directions**: This transgenic model exhibits substantial utility for investigating the molecular mechanisms underpinning TMEM CT‐mediated neurodegeneration. Further studies of the pathways contributing to TMEM‐mediated neurotoxicity are underway. Additionally, these animals will be useful for studying interactions between other proteinopathies including the relationship between TMEM CT, tau, TDP‐43, and amyloid.

The *C. elegans* mutant *pgrn‐1* (tm985) is a null mutation, featuring a 347 base pair deletion (13245/13246–13592/13593) in the *pgrn‐1* GRN‐like gene (obtained from the National BioResource Project, Japan)^41^ was backcrossed to N2 five times as strain CK560.

Strain EG1285 has an integrated *unc‐47p*::GFP transgene expressed in gamma‐aminobutyric acid‐ergic (GABAergic) neurons that clearly marks the cell bodies and axons of ventral cord VD and DD‐type inhibitory motor neurons.^42^ *C. elegans* strains CK3107 (*spop‐1* null), CK3067 (*sut‐6* null), and CK3012 (*sut‐2* null) were previously generated by CRISPR/cas9 mediated genome editing.^43^, ^44^, ^45^ All TMEM106B transgenic strains described here contain integrated transgenes carrying human TMEM106B‐core encoding cDNAs and a *Pmyo‐3*::mCherry coinjection marker. CK2620 and CK2624 carry *snb‐1p*::TMEM106B‐core (TMEM Tg A and TMEM Tg B), while CK2618 and CK2655 carry *snb‐1*p::dendra2hTMEM106b‐core (d‐TMEM Tg C and d‐TMEM Tg D). Double mutants with TMEM106B expression were generated by crossing CK2620 or CK2624 with other strains carrying other transgenes or mutations of interest.

All strains were maintained at 20°C on NGM plates seeded with OP50 *Escherichia coli* .^39^ Genotypes were confirmed by polymerase chain reaction (PCR) and sequencing for all strains with nonobvious phenotypes.

### 2.4 Live *C. elegans* imaging

Live worms were mounted on a 4% agarose pad immobilized with ∼50 mM sodium azide [Sigma] and covered with a glass slip. Representative images were acquired using a Nikon A1R confocal microscope with a 10 × air objective or 40 × or 100 × oil immersion lens (Nikon USA, Melville, NY). Z‐plane stacked images were flattened into a maximum‐intensity projection using ImageJ software.

### 2.5 Immunohistochemistry

Day 1 adult worms were fixed in 1% formaldehyde solution and permeabilized by freeze cracking as described previously.^46^ Fixed and permeabilized animals were counterstained with 300 nM 4,6‐diamidino‐2‐phenylindole (DAPI) nuclear stain in PBS. Fixed whole animals were stained with anti‐TMEM CTF monoclonal antibody (TMEM239)^47^ at a dilution of 1:500 or anti‐phospho‐Tau antibody (PHF‐1)^48^ at a dilution of 1:100. Alexa 488‐conjugated anti‐rabbit antibody (Invitrogen) and ALEXA 647‐conjugated anti‐mouse antibody was used as the secondary antibody at a dilution of 1:1000. Images were acquired by a Nikon A1R confocal microscope with a 100 × oil immersion lens (Nikon USA, Melville, NY) or Andor Dragonfly 200 63 × oil immersion lens (Andor UK, Bellfast) and processed in FIJI:imageJ.

### 2.6 Behavioral analysis

NGM plates of day 1 adult *C. elegans* were flooded with 1 mL of M9 buffer (22 mM KH_2_PO_4_ monobasic, 42.3 mM Na_2_HPO_4_, 85.6 mM NaCl, 1 mM MgSO_4_), and swimming worms were pipetted onto a 35 mm unseeded NGM plate. Approximately 30 s following the addition of the M9 buffer, worms were recorded swimming for 1 min at 14 frames per second. These videos were captured and analyzed with WormLab 2021 (MBF Bioscience). The frequency of body bends, or turns, as defined as a change in body angle of a least 20 from a straight line measured by the quarter points and midpoint of the worms, was quantified as a readout of locomotion. Worms tracked for less than 30 s were omitted from this analysis. At least three independent samples totaling at least 60 worms per strain were counted for every comparison.^49^

### 2.7 Neurodegeneration assays

Worms at larval stage 4 (L4) were selected and moved onto new NGM plates with OP‐50. At 24 h later, live worms were placed on a 2% agarose pad containing 0.01% sodium azide to immobilize the worms. Worms were imaged under fluorescence microscopy and scored for a number of GABAergic neurons. At least three independent samples totaling at least 60 worms per strain were counted for every comparison. Data were analyzed using GraphPad Prism software.^50^, ^51^

### 2.8 Protein extraction

*C. elegans* was grown from hypochlorite‐purified eggs at 20°C for 3 days on 5XPEP plates until young adults. Worms were washed off plates with M9 buffer, collected by centrifugation, and grown further on egg plates as previously described.^52^ Well‐fed worms were collected from egg plates, harvested and isolated via sucrose floatation, washed into M9 buffer, and collected in 250 µL aliquots of packed worms before snap‐freezing in liquid nitrogen to be stored at −70°C. These worm samples were thawed, sonicated (11 × 15 s at 70% power, and put on ice in between sonications) in TM‐LS Buffer (10 mM Tris, 800 mM NaCl, 1 mM EDTA, 10% Sucrose, pH 7.5). Sarkosyl insoluble material was separated from soluble protein through ultracentrifugation (two spins at 140,000 × *g* for 30 mins each). The detergent insoluble material is not readily detectable without rigorous solubilization so a modified Aβ extraction protocol^53^ was utilized to expose TMEM epitopes. Briefly, the aggregated protein was resolubilized by resuspension and sonication in 90% formic acid (FA) to expose TMEM fibrillar epitopes. FA samples were dried in a speedvac to evaporate off FA. Dried protein aggregate pellets were solubilized in 100 µL 5xSDS (46 mM Tris, 5 mM EDTA, 200 mM dithiothreitol, 50% sucrose, 5% sodium dodecyl sulfate, 0.05% bromophenol blue) protein sample buffer immediately after FA treatment. 200 µL aliquots were taken after initial solubilization in TM‐LS buffer for soluble protein fractions and pelleted. Soluble protein was diluted with 60 µL 1 × sodium dodecyl sulfate (SDS) protein sample buffer. Samples were sonicated at 70% for 15 s three times returning to ice between each replicate, boiled for 10 min at 95°C, centrifuged at 13,000 × *g* for 2 min, and stored at −80°C. Samples were loaded (6 µL) onto 4%–15% precast criterion sodium dodecyl sulfate polyacrylamide gel electrophoresis gradient gels and transferred to polyvinylidene fluoride (PVDF) per the manufacturer's protocol (Bio‐Rad). The protein sizing ladder used was Precision Plus Protein Standards (Bio‐Rad). The primary antibodies used were rabbit polyclonal anti‐TMEM106B C‐terminus antibody (TMEM 239)^15^, ^47^ at 1:800 and mouse anti‐tubulin antibody E7 (Developmental Studies Hybridoma Bank) at 1:5000. The secondary antibodies used were anti‐mouse horseradish peroxidase (HRP) or anti‐rabbit HRP (Jackson Immuno Research) at 1:5000. Enhanced chemiluminescence (ECL) substrate was used to visualize the membrane (Bio‐Rad). Chemiluminescence signals were detected with the LiCor Imager and quantified with Fiji.^54^ Insoluble protein samples were also loaded (6 µL) onto 4%–15% precast criterion SDS polyacrylamide gel electrophoresis gradient gels and stained with Coomassie Brilliant Blue (Bio‐Rad) for normalization of insoluble protein fractions imaged by immunoblot.

### 2.9 Longevity measurements

L4 stage worms were selected onto NGM plates and then transferred onto NGM plates with added 5‐fluorodeoxyuridine (FUDR, 0.05 mg/mL) to inhibit the growth of progeny. Worms were housed at 25°C and scored every 1–2 days by gentle touching with a platinum wire. Failure to respond to touch was scored as dead. Worms that died as a result of FUDR or crawled off plates were censored.^55^ Statistical analysis was performed using GraphPad Prism software.

### 2.10 Fluorescence recovery after photoablation (FRAP)

Day 1 adult worms were used for all FRAP experiments and immobilized on 5% agarose pads for imaging, covered in 2 µL of a solution of 500 µM sodium azide in M9 medium. Coverslips were sealed with molten Vaseline. Imaging was performed on a Nikon A1R confocal microscope with a 100 × oil immersion lens (Nikon USA, Melville, NY) which allows for photobleaching of the regions of interest (ROIs). Two images in 2D were taken pre‐bleach, and images were taken every 5 s for 1 min post‐bleach using the 100 × oil immersion lens. Two cells or puncta were bleached for every worm. The FRAP measurement was performed with a 405 nm Galvano laser at a power of 2. Nikon software reported fluorescent intensity for each ROI at every time point. Loss and return of fluorescent intensity were measured and graphed with GraphPad Prism software, and images were processed in FIJI:imageJ.^56^, ^57^, ^58^

### 2.11 Colocalization analysis

Images were taken with an Andor Dragonfly 200 63 x oil immersion lens (Andor UK, Belfast) and processed in FIJI:imageJ. Colocalization analysis was performed with FIJI:imageJ software. For % intensity overlapping, ROIs were generated for TMEM CT intensity and *ctns‐1* intensity, and measurements of total/overlapping intensity were calculated.^59^

## 3 RESULTS

### 3.1 A transgenic *C. elegans* model of TMEM106B proteinopathy

**FIGURE 1:**
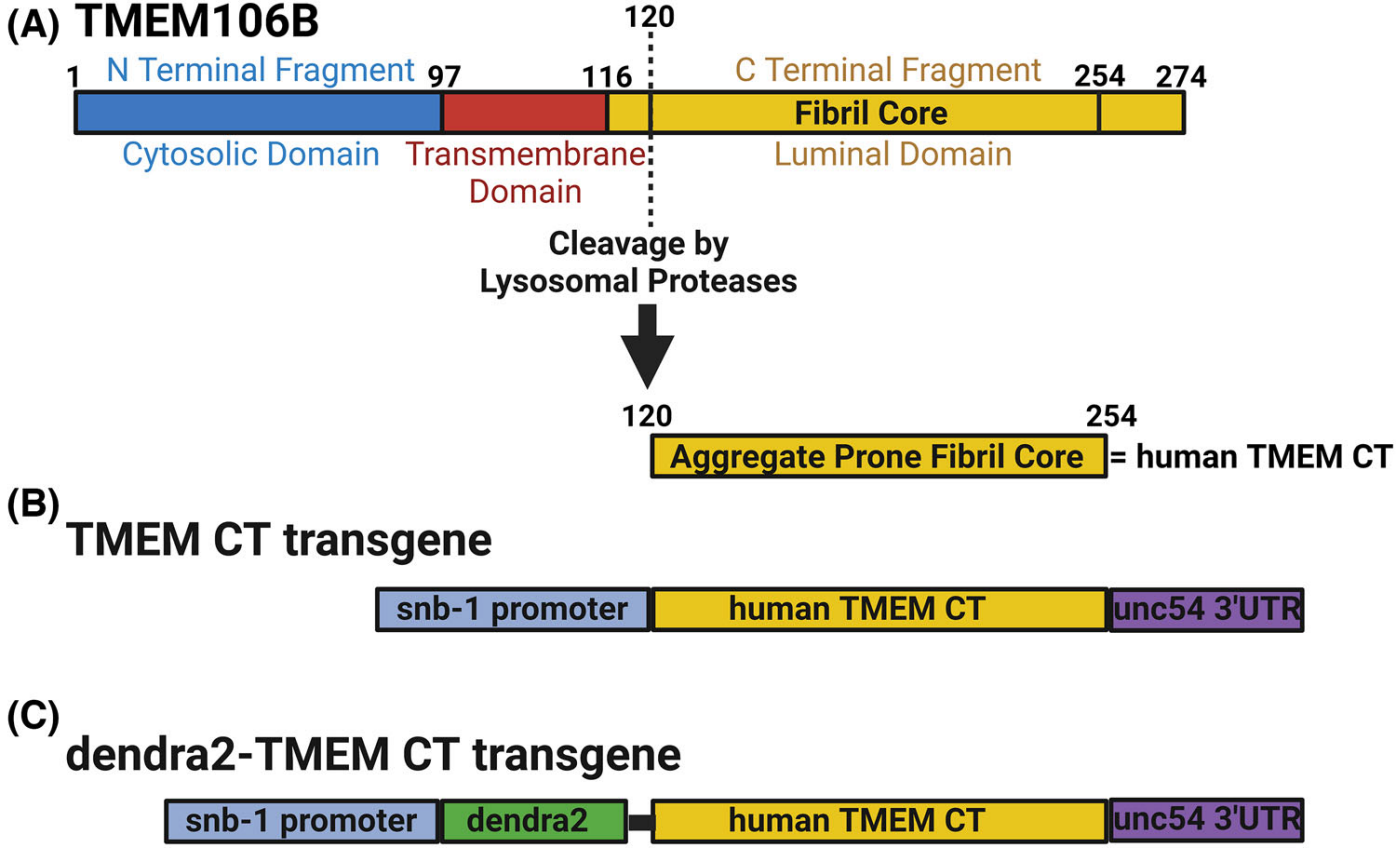
Transgenic insertion of TMEM CT in *Caenorhabditis elegans*. (A) Transmembrane protein TMEM106B contains three domains: the N‐terminal cytosolic domain, the transmembrane domain, and the C‐terminal luminal domain. The luminal domain contains an aggregate‐prone fibril core (TMEM CT; AA 120‐254). Diagram of transgenes containing (B) TMEM CT or (C) dendra2‐tagged TMEM CT (d‐TMEM CT) expressed under the pan‐neuronal *snb‐1* promoter. (D) Experimental flow through for analysis of neuronal degeneration via thrashing assays and counts of GABAergic neurons by use of a fluorescent reporter strain. TMEM CT, TMEM106B C‐terminal fragments.

The TMEM106B protein consists of an N‐terminal cytosolic domain, a single transmembrane domain, and a CT luminal domain. The CT domain, from amino acids 120–254 consists of highly aggregate prone β‐sheets, and makes up the fibril core recently discovered to aggregate in the brain across a number of neurodegenerative diseases (Figure 1A).^12^, ^13^, ^14^, ^15^, ^16^ *C. elegans* constructs expressing the human TMEM CT fragment were designed to model the TMEM CT aggregation seen in a variety of neurodegenerative conditions. We have previously expressed tau or TDP‐43 using a similar strategy to model the neurodegenerative proteinopathy associate with tau and/or TDP‐43 aggregation.^38^, ^40^, ^60^ We generated transgenic *C. elegans* expressing human TMEM CT encoding cDNAs under control of the *snb‐1* promoter (a *C. elegans* pan‐neuronal promoter) (Figure 1B). These transgenic strains are referred to as TMEM Tg A and TMEM Tg B hereafter. We also generated transgenic *C. elegans* expressing dendra2 (a photo‐convertible fluorescent protein) tagged human TMEM CT encoding cDNAs under control of the same pan‐neuronal promoter (hereafter, d‐TMEM CT–Figure 1C) for ease of visualizing TMEM CT localization. These transgenic strains will be referred to as d‐TMEM Tg C and d‐TMEM Tg D.

### 3.2 TMEM CT aggregation promotes neurotoxicity in *C. elegans*

**FIGURE 2:**
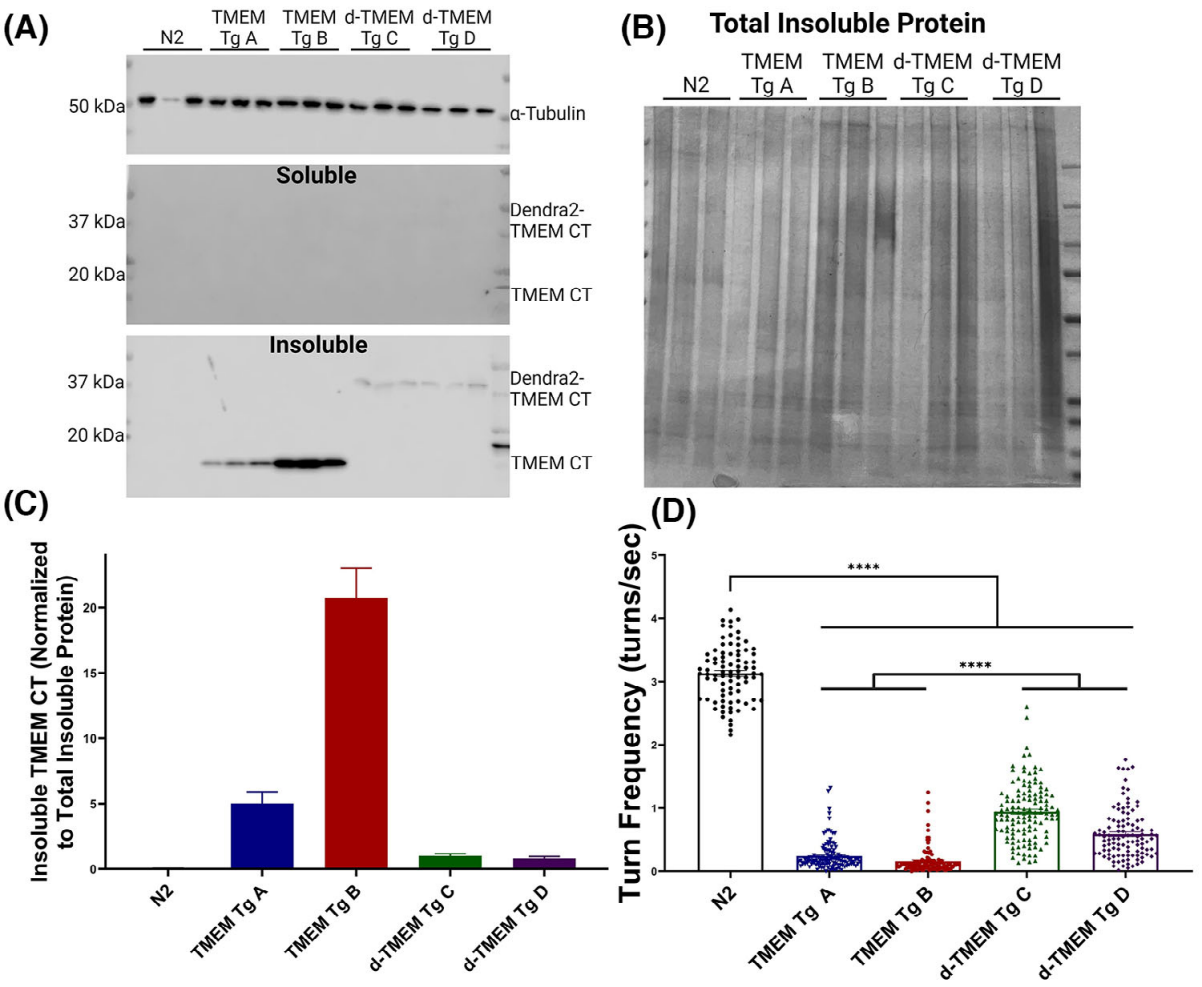
Aggregation of TMEM CT induces motor phenotype in Tg *Caenorhabditis elegans*. (A) Immunoblot analysis using TMEM CT specific antibody TMEM 239 detects TMEM CT (17 kDa) and d‐TMEM CT (42 kDa) in insoluble protein fractions of Tg *C. elegans* strains. No signal was observed in soluble protein fractions, confirming the highly aggregate‐prone nature of this protein fragment. (B) Coomasie Brilliant Blue staining of insoluble protein fractions. (C) Quantification of immunoblot analysis of insoluble protein fractions. Samples were not collected from age‐matched populations. (D) Liquid thrashing assay of TMEM CT and dendra‐TMEM CT strains assessed by computer analysis. *n* > 60, *N* = 3 for each strain. Day 1 adult *C. elegans* expressing both TMEM CT and dendra TMEM CT exhibit significantly impaired thrashing behavior as compared to N2 worms (*p* < 0.0001), indicative of neuronal degeneration. *p*‐Values denoted as **** for *p* < 0.0001, error bars represent standard error of the mean. TMEM CT, TMEM106B C‐terminal fragments.

Protein analysis by immunoblot of transgenic *C. elegans* strains detected no TMEM CT in detergent soluble protein fractions (Figure 2A–C). However, based on extreme TMEM CT detergent insolubility in human disease, TMEM CT may accumulate in *C. elegans* as a highly detergent insoluble protein fragment. Studies with other highly aggregated amyloids have shown FA treatment to be an effective way of solubilizing detergent insoluble protein aggregates. Therefore we treated lysates from TMEM Tg animals with FA to expose aggregated TMEM106B protein epitopes.^53^ Further immunoblot analysis of FA‐extracted protein aggregates confirmed TMEM CT expression (Figure 2A–C), and the evident aggregation propensity of this protein. We observed that d‐TMEM Tg lines visually appeared to have lower levels of TMEM CT aggregation as compared to the TMEM Tg lines.

Next, developmentally staged populations of TMEM CT transgenic *C. elegans* were assayed using motility (thrashing in liquid) as a sensitive readout of neuronal function. At day 1 of adulthood, TMEM CT and d‐TMEM CT expressing lines performed significantly worse than the wild‐type N2 strain using the liquid thrashing assay as measured by observer‐independent digital video analysis (Figure 2D). This neuronal dysfunction was present at an early developmental timepoint, as TMEM and d‐TMEM Tg strains were similarly impaired at the L2 stage of development (Figure S1). At both day one of adulthood, and stage L2 of development, the d‐TMEM Tg strains exhibited a moderate degree of behavioral deficiency compared to the severe impairment in TMEM Tg animals. All detectable TMEM CT appears in the detergent‐insoluble fraction and the abundance of TMEM CT appears related to the extent of behavioral deficits, suggesting a tie between the abundance of TMEM CT aggregates and the severity of neuronal dysfunction. By assessing the behavior of our TMEM CT Tg strains at the L2 stage of development, day 1 of adulthood, and day 4 of adulthood, we observe a progressive loss of neuronal function, indicating the neurodegenerative effects of TMEM CT aggregation progress with age in our model (Figure S2).

**FIGURE 3:**
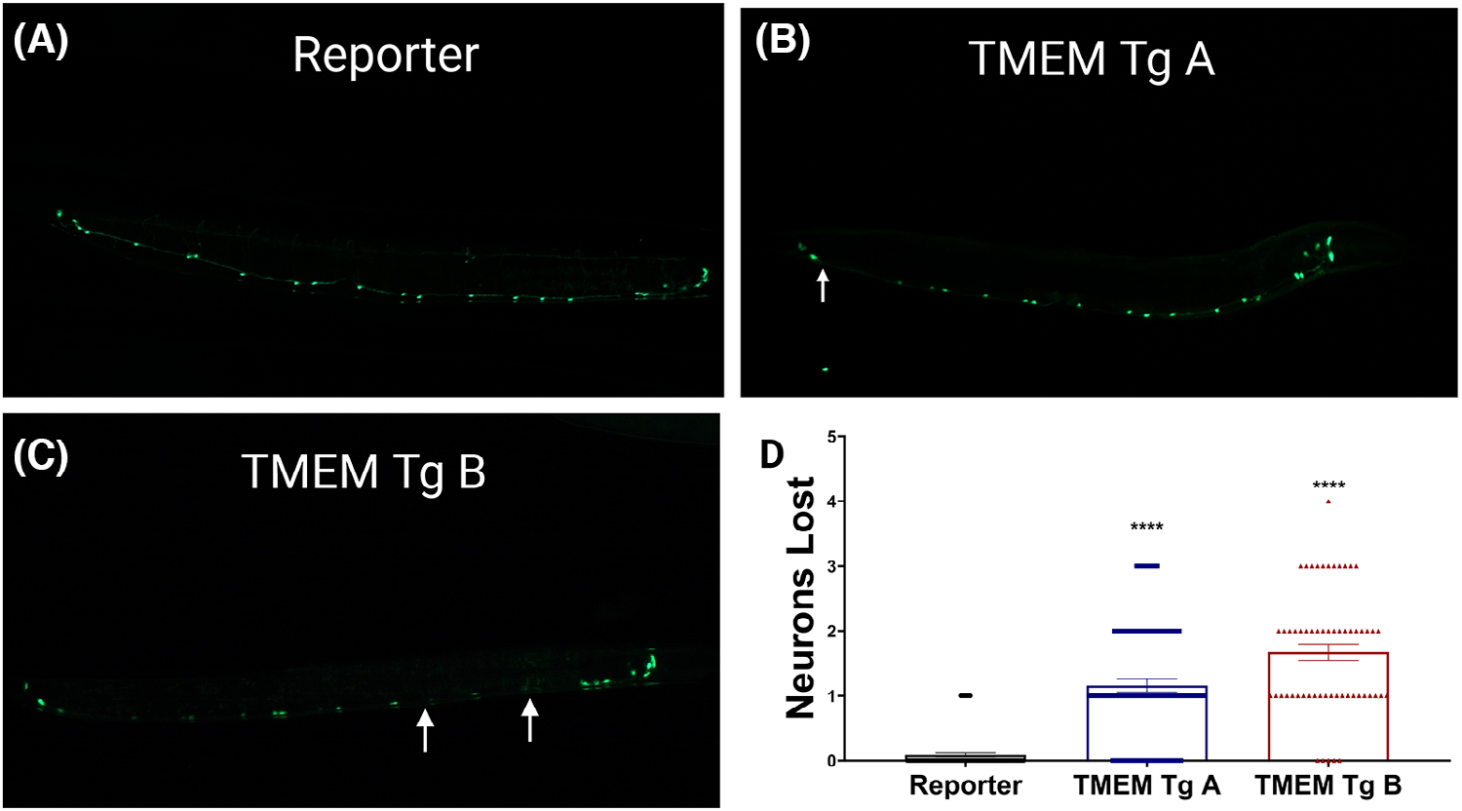
Neurotoxicity of TMEM CT in *Caenorhabditis elegans*. At day 1 of adulthood, TMEM Tg A loses on average 1 of 19 GABAergic neurons along the motor cord, while TMEM Tg B loses nearly 2 of 19 on average as visualized by the *unc‐47p*:GFP reporter (EG1285). Both Tg strains lose significantly more GABAergic neurons than the reporter strain (*p* < 0.0001). (A–C) Representative images for (A) the reporter strain, (B) TMEM Tg A, and (C) TMEM Tg B. Arrows indicate lost neurons. (D) Graphical representation of neuronal counts. *n* > 60, *N* = 3 for each strain. *p*‐Values are denoted as **** for *p* < 0.0001, error bars represent standard error of the mean. TMEM CT, TMEM106B C‐terminal fragments.

To determine if behavioral deficits coincide with neuronal degeneration, we examined the integrity of GABAergic D‐type motor neurons. We crossed TMEM CT lines with a reporter transgenic strain expressing green fluorescent protein (GFP) in GABAergic motor neurons under the control of the *unc‐47* promoter, allowing in vivo assessment of neurons in living TMEM Tg animals. At day 1 of adulthood, TMEM CT expressing strains showed significant loss of GABAergic motor neurons compared to the reporter strain, with TMEM Tg A losing around 1 of 19 neurons and TMEM Tg B losing close to 2 of 19 neurons on average (Figure 3). Given the lack of redundancy within the *C. elegans* nervous system, we consider a ∼10% loss of GABAergic neurons to be substantial. We also crossed d‐TMEM CT Tg lines with a reporter transgenic strain expressing mCherry (a red fluorescent protein) in GABAergic motor neurons under the control of the *unc‐47* promoter and conducted a preliminary analysis of neuronal loss in the d‐TMEM CT Tg strains. At day 1 of adulthood, d‐TMEM CT worms exhibited a significant, but moderate loss of around 0.6 out of 19 neurons on average (Figure S3), supporting our findings of modest neuronal dysfunction and TMEM CT expression. A similar analysis of TMEM Tg strains at the L2 stage of development indicated no detectable loss of GABAergic neurons during development (Figure S4). We take the presence of neuronal dysfunction and lack of neuronal loss at the L2 stage to indicate that neuronal dysfunction precedes neuronal loss.

**FIGURE 4:**
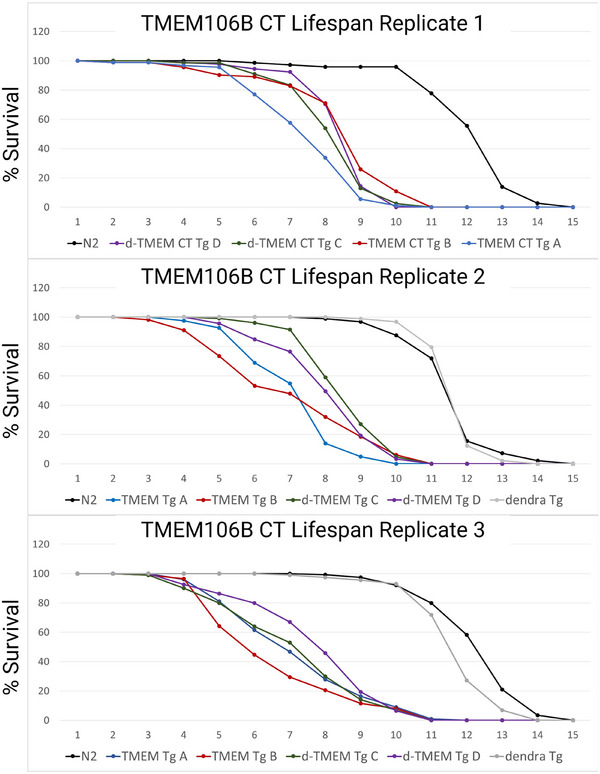
TMEM CT aggregation severely decreases lifespan of *Caenorhabditis elegans*. Lifespan assays of transgenic and wild‐type *C. elegans* under FUDR treatment at 25°C. *n* > 72 worms per strain, *N* = 3. Wild‐type (N2) worms and transgenic worms expressing only dendra2 had a median survival of around 12 days of adulthood. TMEM CT Tg and d‐TMEM CT Tg strains had a significantly reduced median survival ranging from 6 to 8 days of adulthood. FUDR, fluorodeoxyuridine; TMEM CT, TMEM106B C‐terminal fragments.

To assess the impact of TMEM CT aggregation on *C. elegans* longevity, we conducted lifespan analysis on our transgenic strains. Under our conditions, the wild‐type N2 strain, as well as *C. elegans* expressing dendra2 alone had a median survival of around 12 days of adulthood. Our TMEM CT Tg as well as d‐TMEM CT Tg strains had median survivals ranging from 6 to 8 days of adulthood, representing a severe reduction in lifespan (Figure 4 and Table S2).

### 3.3 Localization of TMEM CT Aggregates in *C. elegans*

To visualize TMEM CT expression, we conducted live imaging fluorescent microscopy experiments to observe the organismal location of TMEM CT accumulation. Representative images show the expression of d‐TMEM CT in d‐TMEM Tg animals and dendra2 expression in dendra Tg animals, lacking the TMEM CT (Figure S5). At day 1 of adulthood, both the dendra Tg and the d‐TMEM Tg strains contain cell body‐filling puncta, primarily in the nerve ring and along the nerve cord. However, the d‐TMEM Tg strain exhibits a visibly lower degree of diffuse dendra2 signal, which suggests a very high proportion of aggregated TMEM CT. Representative images of animals at larval stage 2 (L2) show a similar trend, indicating TMEM CT aggregation during worm development (Figure S6).

**FIGURE 5:**
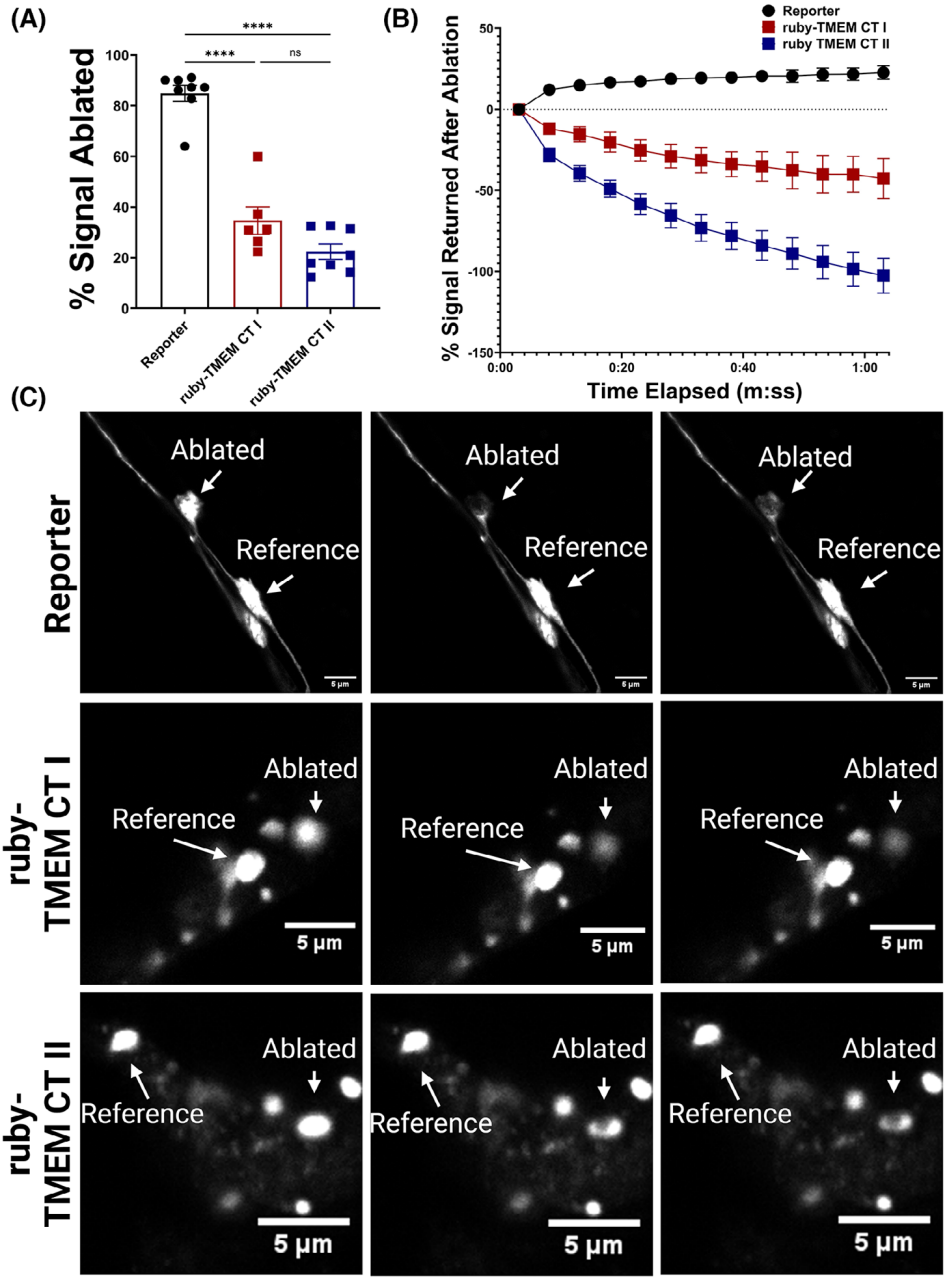
FRAP of ruby‐TMEM CT *Caenorhabditis elegans* at day 1 of adulthood. (A) Observation of % signal reduction by photobleaching indicates that the ruby‐TMEM CT fluorescence is much more resistant to photobleaching than our GFP reporter control (**** represents *p* < 0.00001, *n* = 6‐8 cells, error bars represent standard error of the mean). (B) FRAP analysis shows no recovery of ruby‐TMEM CT fluorescence after photobleaching. (C) 100 × magnification of ablated and reference cells or puncta. The left column represents images before ablation, the middle column represents images immediately after ablation, and the right column represents images 1 min post ablation. Size bar represents 5 µm. Images taken by Nikon Confocal Microscope and processed in FIJI:imageJ. FRAP, fluorescence recovery after photoablation; GFP, green fluorescent protein; TMEM CT, TMEM106B C‐terminal fragments.

To quantify the aggregation state of TMEM CT fragments in our model, we conducted Fluorescence Recovery after Photobleaching (FRAP) on day 1 adult *C. elegans* strains expressing the TMEM CT fragment N‐terminally fused to mRuby3, a red fluorescent protein proven to work well for FRAP experiments.^56^, ^61^ We observed that the ruby‐TMEM fluorescence was less susceptible to photoablation than our reporter, EG1285 expressing diffuse GFP in GABAergic neurons, with only 20%–40% signal loss in our ruby‐TMEM Tg strains compared to the 80% signal reduction in our reporter (Figure 5A). After 1 min of recovery time post ablation, we observed no recovery of the ruby‐TMEM CT fluorescence, indicating no fluidity of ruby‐TMEM CT signal (Figure 5B–C). Together this points to the highly immobile nature of TMEM CT aggregates in our model, corroborating immunoblot data and reflecting what is seen in human disease.

**FIGURE 6:**
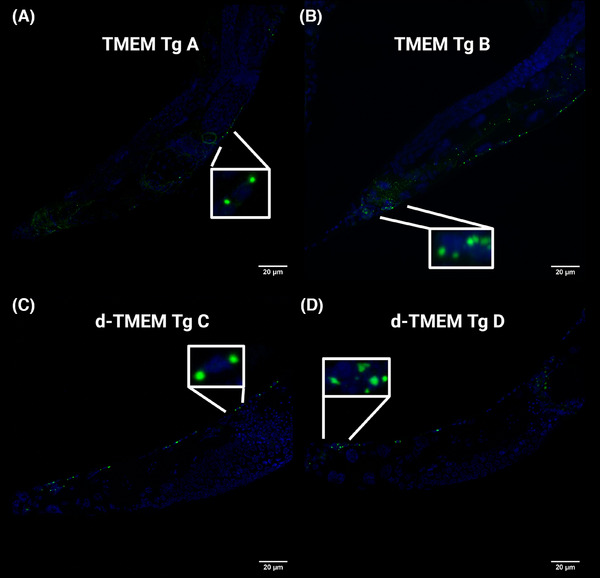
Localization of TMEM CT aggregates. (A,B) 63 × images of TMEM CT immunofluorescence in day 1 adult TMEM CT Tg *Caenorhabditis elegans* along the nerve cord indicate TMEM CT juxtanuclear aggregates. Blue fluorescence indicates cell nuclei (DAPI), and green fluorescence indicates TMEM CT (TMEM 239 rabbit antibody 1:500). (C–D) 63 × images of day 1 adult d‐TMEM CT Tg *C. elegans* indicate d‐TMEM CT aggregates localize in a similar manner to TMEM CT aggregates. Blue fluorescence indicates cell nuclei (DAPI), and green fluorescence indicates d‐TMEM CT. Images taken by Andor Dragonfly 200 Microscope and processed in FIJI:imageJ. DAPI, 4,6‐diamidino‐2‐phenylindole; TMEM CT, TMEM106B C‐terminal fragments.

In attempts to understand where in the cell TMEM CT aggregates are forming, we performed IHC of TMEM Tg strains, with DAPI staining for cell nuclei and an antibody against TMEM CT (TMEM 239),^47^ and similarly DAPI stained d‐TMEM Tg worms. We observed TMEM CT and d‐TMEM CT protein accumulates in the cytoplasm in juxtanuclear aggregates in day 1 adults (Figure 6).

**FIGURE 7:**
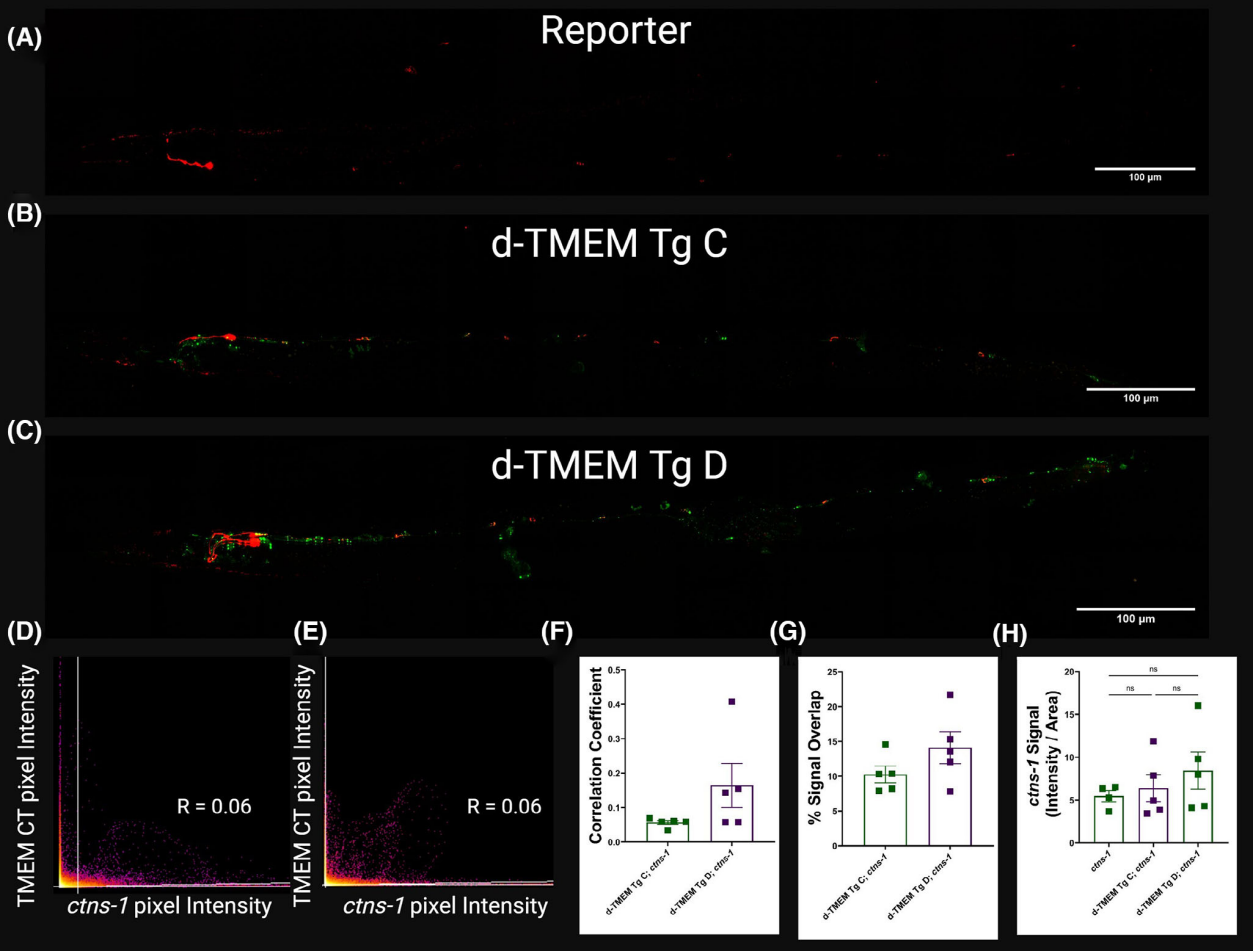
Colocalization analysis of TMEM CT and lysosomal marker *ctns‐1*::mCherry. (A–C) 63 × magnification images of d‐TMEM Tg; *ctns‐1*::mCherrry *Caenorhabditis elegans* with green fluorescence indicating d‐TMEM CT and red fluorescence indicating *ctns‐1*. Size bars indicate 100 µm. (D–F) Representative scatter plots of d‐TMEM CT pixel intensity against *ctns‐1* pixel intensity for d‐TMEM CT C and d‐TMEM CT D, respectively. Average Pearson correlation coefficients of < 0.1 to < 0.2 indicate weak to minimal colocalization of d‐TMEM CT and *ctns‐1* (*n* = 5 for each strain). (G) Assessment of % signal overlap showed that roughly 10%–14% of TMEM CT intensity overlapped with *ctns‐1*::mCherry signal (*n* = 5 per strain). (H) Analysis of *ctns‐1*::mCherry signal intensity indicates no significant impact of d‐TMEM CT expression on *ctns‐1*::mCherry intensity (ns represents *p* > 0.05, error bars represent standard error of the mean). TMEM CT, TMEM106B C‐terminal fragments.

We generated a cross between our d‐TMEM Tg strains and a lysosomal reporter strain with the lysosomal protein, cystinosin homolog (CTNS‐1), C‐terminally fused to the red fluorescent protein, mCherry. Live imaging of these worms showed d‐TMEM CT aggregates often formed adjacent to lysosomal signal, but did not appear to be engulfed by lysosomes (Figure 7A–C). Further analysis of potential d‐TMEM CT and *ctns‐1* colocalization generated average Pearson correlation coefficients of < 0.1 for d‐TMEM CT C; *ctns‐1* and < 0.2 for d‐TMEM CT D; *ctns‐1*, indicating negligible to weak colocalization. Similarly, we observed that only 10%–14% of TMEM CT intensity overlapped with *ctns‐1* (Figure 7D‐G). Assessment of total *ctns‐1* fluorescent intensity between the *ctns‐1* reporter and the d‐TMEM Tg; *ctns‐1* doubles indicate that aggregation of TMEM CT does not significantly impact *ctns‐1* intensity (Figure 7H). From this analysis, we do not observe any direct interaction between TMEM CT aggregates and lysosomes.

### 3.4 Lack of genetic interaction of TMEM Tg with progranulin loss of function in *C. elegans*

SNPs in the human TMEM106B gene confer risk of development and severity for a number of neurodegenerative diseases, but most significantly in cases of FTLD‐TDP caused by PGRN haplo‐insufficiency.^17^, ^18^ Given that PGRN is a lysosomal protein governing protease activity, and partial loss of PGRN leads to impaired lysosomal function,^62^, ^63^, ^64^ we expect that loss of PGRN is involved in the mechanisms through which the TMEM CT ceases to be degraded and begins aggregation in disease.

**FIGURE 8:**
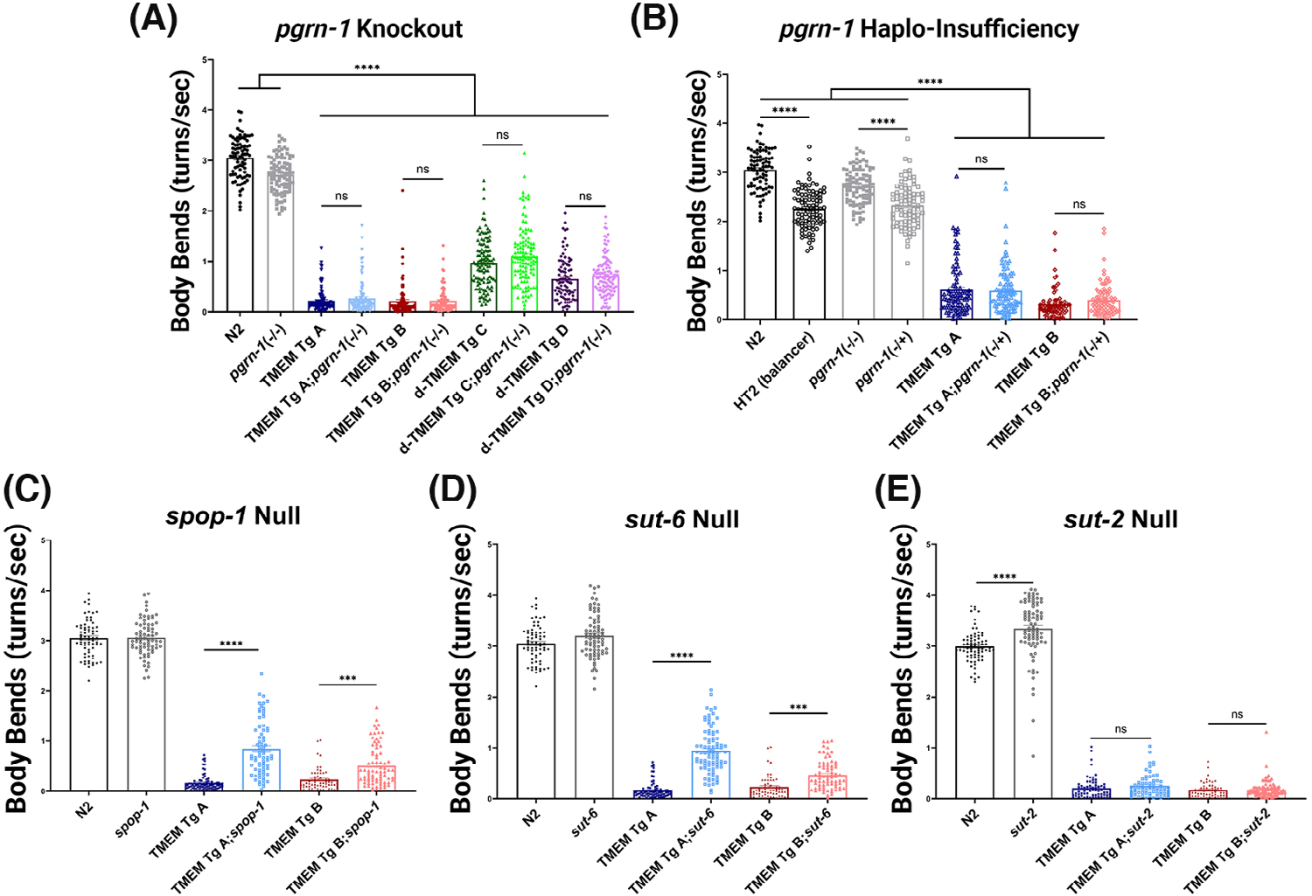
Potential modifiers of behavior for TMEM CT *Caenorhabditis elegans* (A) Liquid thrashing assay of TMEM CT strains with complete loss of *pgrn‐1* assessed by computer analysis. *n* > 60, *N* = 3 for each strain. Complete loss of *pgrn‐1* had no significant impact on worm behavior. (B) Liquid thrashing assay of TMEM CT expressing strains with partial loss of *pgrn‐1* assessed by computer analysis. *n* > 60, *N* = 3 for each strain. Similarly, partial loss of *pgrn‐1* had no significant impact on worm behavior. (C) Liquid thrashing assay of TMEM CT expressing strains with complete loss of SPOP‐1 assessed by computer analysis. *n* > 60, *N* = 3 for each strain. Loss of *spop‐1* confers a weak, but statistically significant return of function for TMEM CT expressing strains. (D) Liquid thrashing assay of TMEM Tg strains with complete loss of *sut‐6* assessed by computer analysis. *n* > 60, *N* = 3 for each strain. Loss of *sut‐6* confers a weak, but statistically significant return of function for TMEM CT expressing strains. (E) Liquid thrashing assay of TMEM Tg strains with complete loss of *sut‐2* assessed by computer analysis. *n* > 60, *N* = 3 for each strain. Loss of *sut‐2* had no significant effect on mobility of TMEM CT expressing strains. *p*‐Values noted as ns for no significance or **** for *p* < 0.0001, error bars represent standard error of the mean. All behavioral analysis was performed on day 1 adults.

To test this hypothesis, we crossed our TMEM Tg strains with *pgrn‐1* null strains, and analyzed behavioral changes on TMEM Tg/ *pgrn‐1* (‐/‐) strains. We found that complete loss of *pgrn‐1* had no significant impact on behavior of either TMEM Tg strain in day 1 adults (Figure 8A). It is important to note that FTLD involves haplo‐insufficiency of PGRN rather than a full knock out. A previous study showed that, in *C. elegans*, complete loss of *pgrn‐1* did not synergistically impact behavior of Tg TDP‐43 mutant strains, while heterozygous loss of *pgrn‐1* significantly impaired behavior in the same Tg strain.^62^

We generated *pgrn‐1* heterozygotes by crossing *pgrn‐1* null and TMEM Tg animals with the balancer hT2, a homozygous inviable balancer for *C. elegans* chromosomes I and III. Unlike the impact of heterozygous *pgrn‐1* on behavior in a Tg TDP‐43 model, at day 1 of adulthood, we observed no significant effect of *pgrn‐1* haplo‐insufficiency on TMEM Tg behavior with TMEM Tg;*pgrn‐1*(‐/+) worms performing similarly to TMEM Tg strains (Figure 8B).

### 3.5 Interactions of TMEM with common suppressors of tau pathology

TMEM106B pathology co‐occurs with tau pathology in PSP, PD, FTLD‐tau, and AD.^12^, ^13^, ^14^, ^15^, ^16^ In addition, TMEM106B mutation or loss of function can influence tau pathology in transgenic mice.^65^, ^66^ Furthermore, initial results indicate TMEM CT aggregation increases levels of phosphorylated tau (p‐Tau) in a low expression model of tau pathology (Figure S7). Our previous studies using forward genetic screens to isolate suppressors of tau pathology in tau Tg *C. elegans* have revealed a number of modifier genes, including *spop‐1*, *sut‐2*, and *sut‐6*.^43^, ^44^, ^49^, ^67^, ^68^, ^69^, ^70^ To determine whether there are shared mechanisms of neurotoxicity between tau and TMEM CT, we tested whether loss of *spop‐1, sut‐2*, or *sut‐6* might have an effect on the phenotype of our TMEM Tg model at day 1 of adulthood.

TMEM Tg A, *spop‐1*, and TMEM Tg B; *spop‐1* performed significantly better on the thrashing assay than TMEM Tg A and TMEM Tg B alone (Figure 8C). However, with only a 23.6 and 9.8% rescue of phenotype, respectively, we do not consider *spop‐1* a strong suppressor of TMEM CT. Similarly, TMEM Tg A, *sut‐6*, and TMEM Tg B; *sut‐6* performed significantly better than TMEM Tg A and TMEM Tg B (Figure 8D), but only showed a 26.8 and 8.4% return of function, respectively. Lastly, loss of *sut‐2* did not result in any significant modification of phenotype for either TMEM Tg strain (Figure 8E). For comparison, loss of function mutations in *spop‐1, sut‐6, and sut‐2* in our *C. elegans* models of tauopathy resulted in return of functions ranging from 50% to 100% in the same liquid thrashing assay.^43^, ^44^, ^67^

## 4 DISCUSSION

Over the past decade SNPs in TMEM106B have emerged as a reproducible genetic risk factor for development and severity of neurodegenerative diseases, especially FTLD‐TDP.^17^, ^18^, ^19^, ^21^, ^22^, ^23^, ^25^, ^26^, ^27^, ^28^, ^29^, ^30^ Previous studies exploring the role of TMEM106B in neurodegeneration have focused primarily on overall levels of protein expression or its role in lysosomal function. TMEM106B expression can be reduced in the brains of AD patients,^26^ and some studies have shown that loss of TMEM106B can induce severe lysosomal abnormalities in neurodegenerative models, especially in the context of PGRN loss.^21^, ^36^, ^65^ Conversely, TMEM106B reduction has rescues lysosomal function caused by deletion of PGRN^28^, ^71^ and risk associated TMEM106B SNPs may alter chromatin architecture, increasing TMEM106B expression, and induce toxic lysosomal defects.^23^, ^32^, ^35^ Due to the conflicting nature of these findings, the role of TMEM106B in neurodegeneration remains unclear.

Recently, the CT fragment of TMEM106B was demonstrated to form amyloid fibrils in the brains of patients across a diverse range of neurodegenerative diseases including AD and many ADRDs.^12^, ^13^, ^14^, ^16^, ^72^ Furthermore, aggregation of this protein fragment has been observed in the brain naturally with age.^15^ While the relative toxicity of the accumulating TMEM106B CT aggregates remains uncertain, the presence of TMEM106B amyloid across a variety of neurodegenerative diseases indicates involvement in multi‐proteinopathy driven neurodegeneration. Understanding the aggregation propensity of TMEM106B as well as the neurodegenerative potential of TMEM106B fibrillization requires new animal and cellular models of the TMEM106B proteinopathy occurring in aging related neurodegenerative disease.

In this study, we describe a series of new transgenic *C. elegans* lines used to model TMEM106B proteinopathy by expressing TMEM CTs in all neurons. We highlight this work because it represents the first live organism model of TMEM CT aggregation in neurons and recapitulates key aspects of TMEM CT associated neurodegenerative change. The TMEM106B transgene expresses a 135 amino acid CT fragment present in the fibrillar core of TMEM106B amyloid that is predicted to have a high β‐sheet strand content with high aggregation propensity.^12^ Immunoblot analysis of TMEM106B transgenic lines confirmed that when the TMEM CT is expressed in *C elegans*, the protein fragment can only be detected in the detergent insoluble fraction. This, combined with the requirement for rigorous aggregate solubilization using FA to permit TMEM CT detection speaks to the highly aggregated state of TMEM106B expressed in *C. elegans*. *C. elegans* do not contain their own homolog of TMEM106B, and as such it is unknown if *C. elegans* will process full length TMEM106B into fragments in similar manners as in mammalian systems. One common post translational modification of TMEM106B, including on the CT domain, is glycosylation.^16^ The expected sizes of our expressed protein fragments are 17 kDa for the CT fragment alone, and 42 kDa for dendra2 fused to the TMEM CT. Based on the 17 kDa size of the TMEM CT and 42 kDa size of d‐TMEM CT on Immunoblot, these protein fractions did not appear to be glycosylated in our model, although the necessity of FA extraction to solubilize TMEM106B CTF aggregates may mask the endogenous glycosylation state of the proteins. Fluorescent analysis of the TMEM CT Tg model shows cytoplasmic aggregation of this protein fragment, representing a successful attempt to model disease conditions. Behavioral analysis of day 1 TMEM Tg adult animals revealed profound behavioral abnormalities indicative of neuronal dysfunction and possible neurodegeneration. This was corroborated by counts of GABAergic motor neurons, which revealed TMEM CT Tg strains exhibited consistent loss of GABAergic neurons. While loss of an average of two neurons per animal at day 1 of adulthood may seem minor, it represents a loss of more than 10% of this nonredundant neuronal population consistent with the manifestation of behavioral phenotypes. Lifespan analysis of TMEM CT Tg strains indicated a severe decline in lifespan as a result of TMEM CT aggregation. FRAP analysis also demonstrated the highly aggregated nature of TMEM CT fragments in our model, supporting immunoblot data. Taken together these data clearly demonstrate the high aggregation propensity of the TMEM106B CTF and support a strongly neurotoxic character of these aggregates. Furthermore, analysis of transgenic *C. elegans* at the L2 stage of development show TMEM CT aggregation along with evidence of neuronal dysfunction that precedes loss of neurons, as no GABAergic neurons were lost at the L2 stage. Analysis of behavior from the L2 stage of development to day 4 of adulthood demonstrated that TMEM CT driven neuronal dysfunction progresses with age. Thus, the CT domain of TMEM106B should be considered a pathogenic protein, and the concept of TMEM106B proteinopathy warrants further analysis both in the context of age‐related changes to TMEM106B and multi‐proteinopathy disorders.

To explore potential genetic interactions between TMEM106B and PGRN in *C. elegans*, we explored the consequences of *pgrn‐1* loss of function on TMEM CT proteinopathy related phenotypes. TMEM106B SNPs have been seen to have the most significant impacts on risk and disease severity in cases involving PGRN haplo‐insufficiency,^17^, ^18^, ^19^ and alterations in TMEM106B expression can exacerbate lysosomal abnormalities caused by loss of PGRN.^17^, ^18^, ^19^, ^21^, ^25^, ^28^, ^35^, ^36^, ^71^ We expect lysosomal dysfunction induced by loss of PGRN impairs TMEM106B processing, resulting in aggregation and TMEM106B proteinopathy. In order to test this hypothesis, we generated TMEM Tg strains with either full or partial loss of *pgrn‐1* and assessed behavioral readouts. We saw no evidence of *pgrn‐1* modification of behavioral abnormalities induced by TMEM106B proteinopathy caused by either full or partial loss of *pgrn‐1*, indicating no interaction between *pgrn‐1* and TMEM106B in this model system. We attribute this negative finding to the fact that all detectable TMEM CT expressed appears to be aggregated. If loss of PGRN instigates TMEM106B proteinopathy by impairing the lysosome's ability to process this protein fragment, our model expressing a highly aggregation prone fragment bypasses the need for altered lysosomal processing by PGRN loss of function. This would result in no observed alteration in TMEM proteinopathy related phenotypes. Mammalian animal model systems may be helpful in fully recapitulating the influence of PGRN loss of function on TMEM106B processing.

In our previous work on proteinopathy disorders, we generated transgenic *C. elegans* models for tau and TDP‐43 proteinopathy that exhibit robust neuronal dysfunction driven by protein aggregation leading to neurodegeneration. We have uncovered potent genetic modifiers of tau proteinopathy including SPOP‐1, SUT‐2/MSUT‐2, and SUT‐6/NIPP1. Genetic loss of function in these genes strongly suppresses the tau proteinopathy phenotypes evident in transgenic *C. elegans*.^43^, ^44^, ^49^, ^67^, ^68^, ^69^, ^70^, ^73^ Consequently, we investigated whether loss of these strong regulators of tau proteinopathy might impact pathological TMEM106B. Our analysis revealed only modest rescue of TMEM106B proteinopathy phenotypes by *spop‐1* or *sut‐6* loss of function. Furthermore, *sut‐2* loss of function exhibited no rescue of TMEM106B proteinopathy. With little to no rescue, we do not consider any of these genes as effective suppressors of TMEM106B pathology. This may suggest that TMEM106B proteinopathy involves unique mechanisms of neurotoxicity as compared to tau.

Emerging evidence indicates multi‐proteinopathy appears to be a much more common driver of neurodegeneration than previously thought, and understanding how each proteinopathy interacts with the others will prove crucial for developing targeted therapeutic strategies.^8^, ^9^, ^10^, ^11^, ^60^ Having shown the neurotoxic potential of TMEM106B in transgenic *C. elegans*, exploring the interplay between TMEM106B and other common proteinopathies appears the logical next step in investigating the molecular basis of multiple etiology dementia.

## AUTHOR CONTRIBUTIONS

Ruben Riordan performed experiments, analyzed data, edited the manuscript, and wrote the first draft manuscript. Aleen Saxton performed experiments, provided methodology, generated strains, and editing the manuscript. Marina Han performed experiments and edited the manuscript. Pamela J. McMillan performed experiments and edited the manuscript. Rebecca L. Kow provided methodology, analyzed data, and edited the manuscript. Nicole F. Liachko provided methodology, analyzed data, and edited the manuscript. Brian C. Kraemer conceived and oversaw the study, analyzed data, and wrote the manuscript.

## CONFLICT OF INTEREST STATEMENT

The authors have no competing interests to declare. Author disclosures are available in the Supporting Information.

## ETHICS STATEMENT

This study has no human or mammalian data.

## CONSENT FOR PUBLICATION

All authors approve of this manuscript and consent to publication.

## Supporting information



Supporting Information

Supporting Information

## Data Availability

All data supporting the conclusions of this article are included within the article and in additional files provided.
