# Supplementary material for: TMEM106B C‐terminal fragments aggregate and drive neurodegenerative proteinopathy in transgenic Caenorhabditis elegans
Source: Alzheimers Dement. 2024 Dec 23;21(2):e14468. doi: 10.1002/alz.14468 (PMC11848199; doi:10.1002/alz.14468)
Supplement: Supplementary file 1 — Supporting Information [file ALZ-21-e14468-s001.docx]

**Supplementary Materials**

**
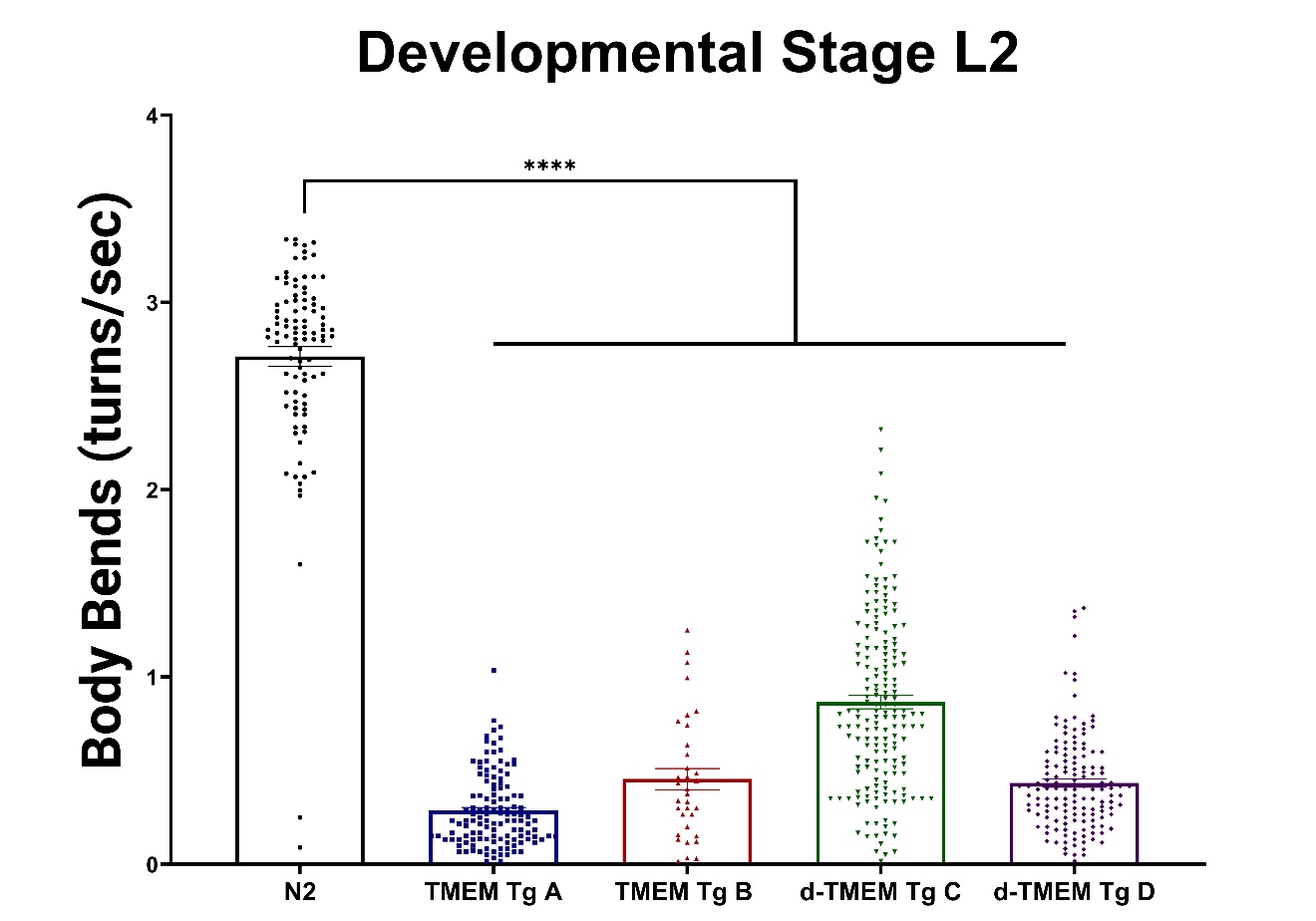
 Supplemental Figure S1. TMEM CT expression impairs locomotion at the L2 stage of development.** Liquid thrashing assay of TMEM CT and d-TMEM CT strains assessed by computer analysis, mean of replicate averages. n > 34, N = 3 for each strain. *C.* *elegans* expressing both TMEM CT and d-TMEM CT exhibit significantly impaired thrashing behavior as compared to N2 worms (p<0.0001), indicative of neuronal degeneration. P values denoted as **** for p<0.0001, error bars represent SEM.


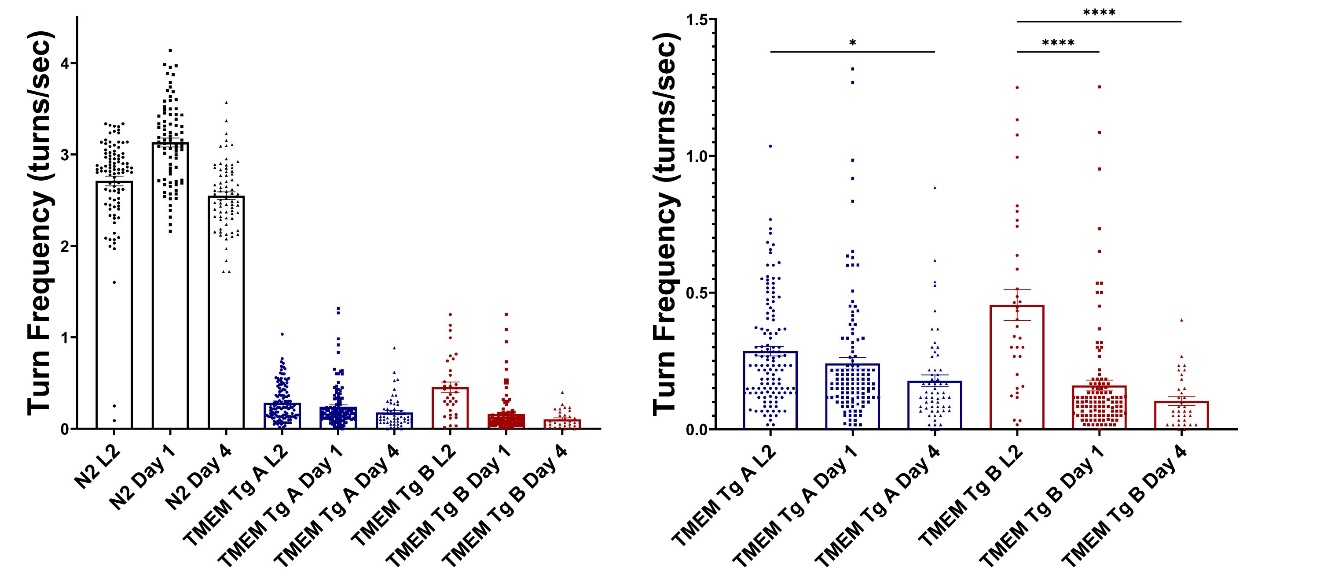
 **Supplemental Figure S2. TMEM CT Expression Impairs Locomotion Progressively With Age.** Liquid thrashing assay of TMEM CT strains at the L2 stage of development, Day 1 of adulthood, and Day 4 of adulthood assessed by computer analysis, mean of replicate averages. n > 34, N = 3 for each strain. *C.* *elegans* expressing both TMEM CT exhibit progressively impaired thrashing behavior, indicative of neuronal degeneration. P values denoted as **** for p<0.0001, and * for p<0.05, error bars represent SEM.


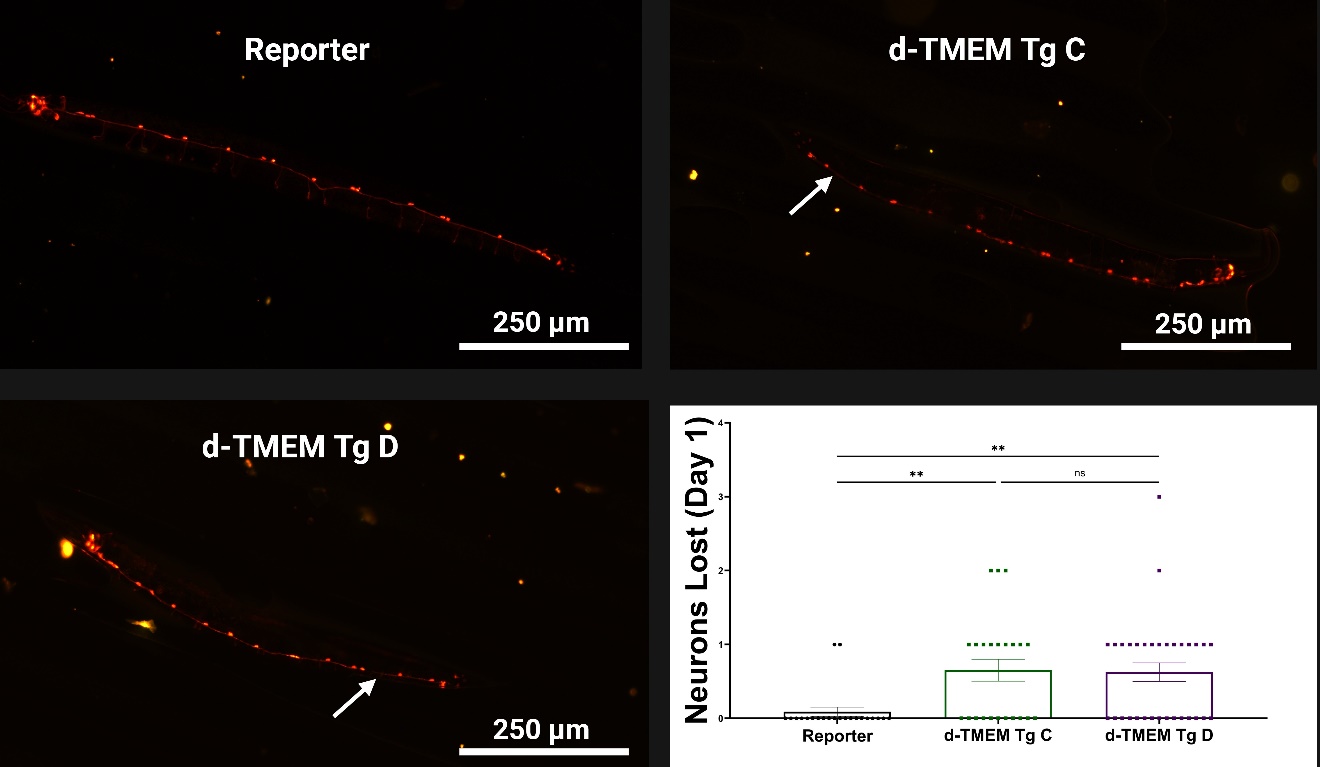
 **Supplemental Figure S3. d-TMEM CT Tg Neuronal Loss at Day 1 of Adulthood.** At Day 1 of adulthood, d-TMEM Tg C and d-TMEM Tg D strains lose on average 0.65 and 0.62 out of 19 GABAergic neurons as visualized by the *unc-47*::mCherry reporter (CK4112). Representative images for the reporter strain, d-TMEM Tg C, and d-TMEM Tg D and graphical representation of neuronal counts. n > 22 for each strain. Size bar indicates 250 µM, and error bars represent SEM. P values denote ** for p<0.01 and ns for no significance.


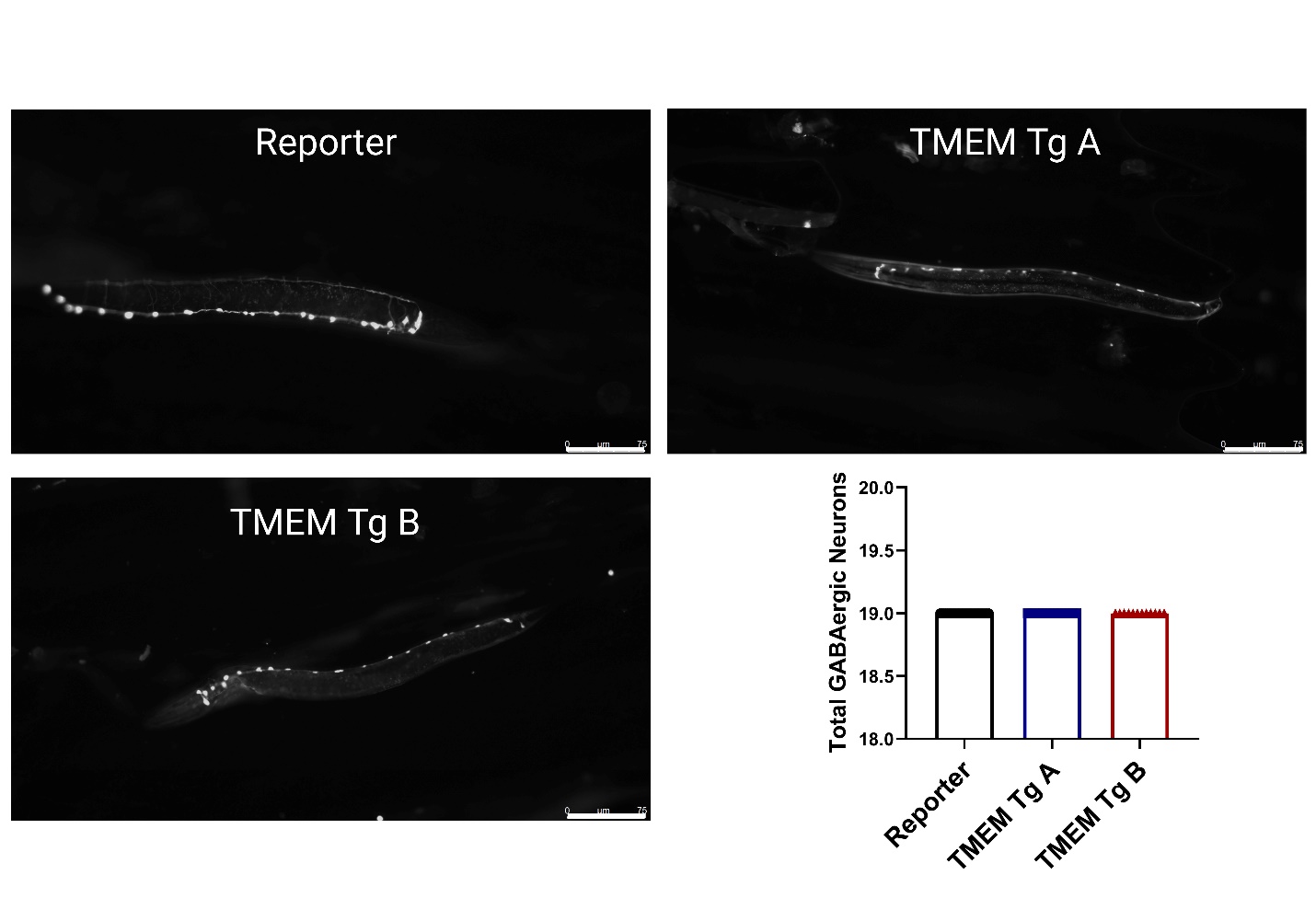
**Supplemental Figure S4. TMEM CT Aggregation Does Not Induce Neuronal Loss in L2 Stage *C. elegans*.** At the L2 stage of development, neither TMEM Tg A nor TMEM Tg B strain lose any of their 19 GABAergic neurons as visualized by the *unc-47*::GFP reporter (EG1285). Representative images for the reporter strain, TMEM Tg A, and TMEM Tg B and graphical representation of neuronal counts. n > 12 for each strain. Size bar indicates 75 µM, and error bars represent SEM.

**
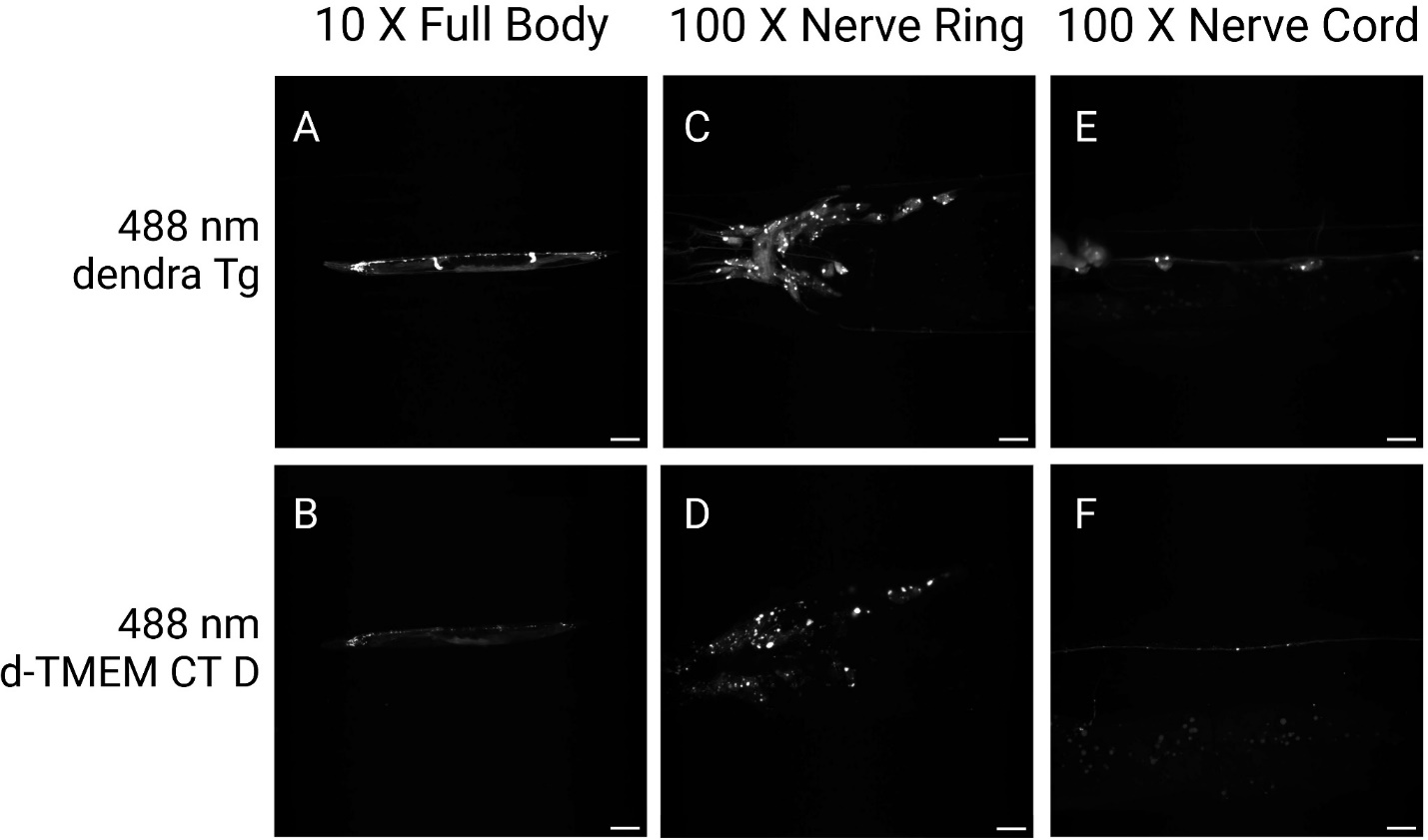
Supplemental Figure S5. Transgenic *C*. *elegans* model expressing d-TMEM CT.** 10X and 100X magnification images of day 1 adult dendra Tg and d-TMEM CT Tg *C*. *elegans*. **A-B)** 10X images of full worm body, signal indicating expression of the dendra2 fluorescent protein. Scale bar represents 100 µm. **C-D)** 100X images of nerve ring, signal indicating expression of the dendra2 fluorescent protein. Scale bar represents 10 µm. **E-F)** 100X images of nerve cord, signal indicating expression of the dendra2 fluorescent protein. Scale bar represents 10 µm.

**
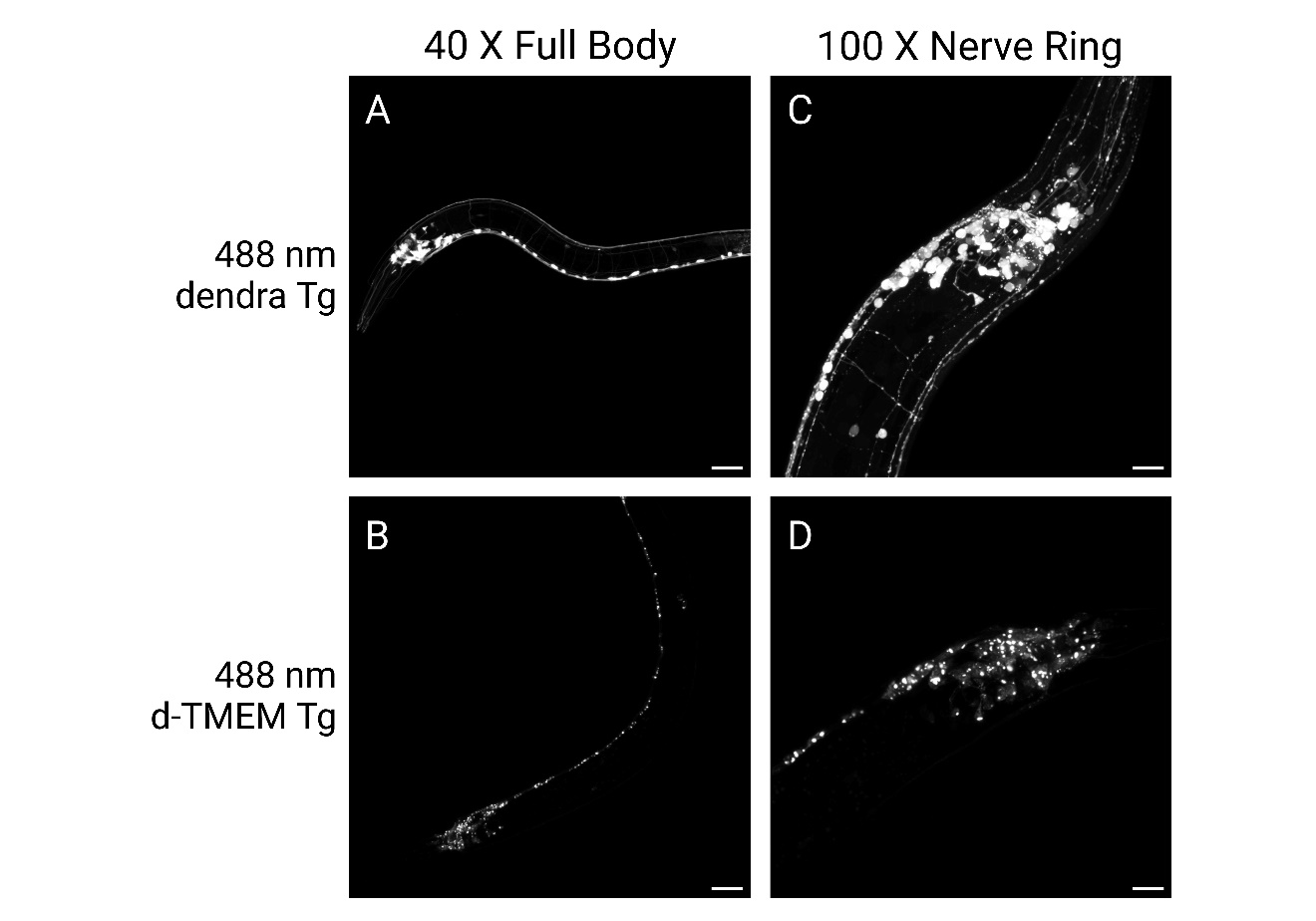
 Supplemental Figure S6. Transgenic *C*. *elegans* model expressing d-TMEM CT at L2 stage of development.** 40X and 100X magnification images of L2 staged dendra Tg and d-TMEM CT Tg *C*. *elegans*. **A-B)** 40X images of full worm body, signal indicating expression of the dendra2 fluorescent protein. Scale bar represents 25 µm. **C-D)** 100X images of nerve ring, signal indicating expression of the dendra2 fluorescent protein. Scale bar represents 10 µm.


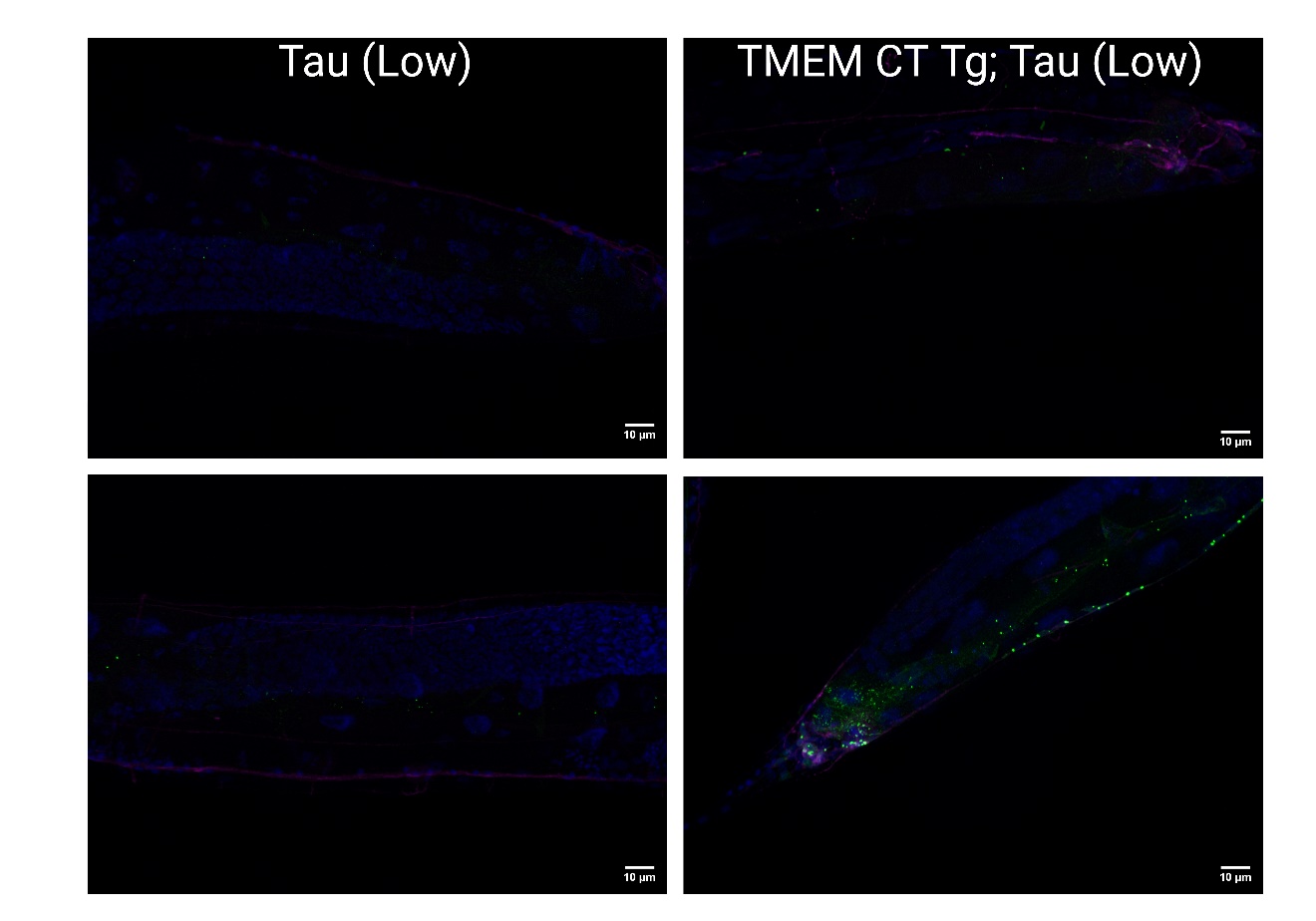


**Supplemental Figure S7. TMEM CT Aggregation Increases Levels of p-Tau in Tau Tg *C. elegans.*** 63X confocal images of TMEM CT and p-Tau immunofluorescence in day 1 adult Tau (Low) and TMEM CT Tg; Tau(Low) expressing *C. elegans* indicate TMEM CT aggregation increases levels of tau phosphorylation in Tau Tg worms*.* Blue fluorescence indicates cell nuclei (DAPI), green fluorescence indicates TMEM CT (TMEM 239 rabbit antibody 1:500), and magenta fluorescence indicates p-Tau (PHF-1 mouse antibody 1:100). Images taken by Andor Dragonfly 200 Microscope and processed in FIJI:imageJ.

| Strain | Genotype | Marker | Description | Abbreviation |
| --- | --- | --- | --- | --- |
| N2 | Wild type | none | Wild type | N2 |
| CK2620 | *snb-1*p::hTMEM106b-core | *myo-3*p::mCherry | TMEM CT Tg | TMEM Tg A |
| CK2624 | *snb-1*p::hTMEM106b-core | *myo-3*p::mCherry | TMEM CT Tg | TMEM Tg B |
| CK2618 | *snb-1*p::dendra2hTMEM106b-core | None | d-TMEM CT Tg | d-TMEM Tg C |
| CK2655 | *snb-1*p::dendra2hTMEM106b-core | None | d-TMEM CT Tg | d-TMEM Tg D |
| CK2565 | *snb-1*p::dendra2 | None | dendra2 Tg | dendra Tg |
| EG1285 | *lin-15B&lin-15A(n765)* oxIs12 X | *unc47*p::GFP | GABAergic Reporter | Reporter |
| CK560 | *pgrn-1(tm985)* | None | *pgrn-1 null* | *pgrn-1* |
| hT2 | bli-4(e937) let-?(q782) qIs48) | *myo-2*p::GFP | Chromosome I and III balancer | HT2 |
| CK3107 | *spop-1(bk3107)* | None | *spop-1* null | *spop-1* |
| CK3067 | *sut-6(bk3067)* | None | *sut-6* null | *sut-6* |
| CK3012 | *sut-2(bk3012)* | None | *sut-2* null | *sut-2* |
| KG2430 | *unc-129*p::*ctns-1*::mCherry + *nlp-21*p::Venus + *ttx-3*p::RFP | None | Lysosomal Reporter | *ctns-1* |
| CK1044 | *aex-3p*::Tau WT (4R1N) | *myo-2*p::GFP | Low expressing WT Tau Tg | *tau(Low)* |
| CK4008 | *snb-1*p::mRubyTMEM106b-core | none | ruby-TMEM CT Tg | ruby-TMEM CT I |
| CK4009 | *snb-1*p::mRubyTMEM106b-core | none | ruby-TMEM CT Tg | ruby-TMEM CT II |
| CK4112 | wpIs36 [*unc-47*p::mCherry] | *unc-47*p::mCherry | GABAergic Reporter | Reporter |

**Supplemental Table 1. Strain List.** List of *C.* *elegans* strains, genotypes, markers, descriptions, and abbreviations.

| Rep 1 (1/24-2/8) |  |  | | |  |  |  |  |  |  | |  | |  | |  |  | |  |  |  |
| --- | --- | --- | --- | --- | --- | --- | --- | --- | --- | --- | --- | --- | --- | --- | --- | --- | --- | --- | --- | --- | --- |
| Deaths/day | 1 | 2 | 3 | 4 | 5 | 6 | 7 | 8 | 9 | | 10 | | 11 | | 12 | | | 13 | | 14 | 15 |
| N2 | 0 | 0 | 0 | 0 | 0 | 1 | 1 | 1 | 0 | | 0 | | 13 | | 16 | | | 30 | | 8 | 2 |
| d-TMEM CT Tg D | 0 | 0 | 0 | 1 | 1 | 3 | 2 | 20 | 51 | | 13 | | 0 | | 0 | | | 0 | | 0 | 0 |
| d-TMEM CT Tg C | 0 | 0 | 0 | 1 | 0 | 6 | 6 | 23 | 32 | | 8 | | 2 | | 0 | | | 0 | | 0 | 0 |
| TMEM CT Tg B | 0 | 1 | 0 | 3 | 5 | 1 | 6 | 11 | 42 | | 14 | | 10 | | 0 | | | 0 | | 0 | 0 |
| TMEM CT Tg A | 0 | 1 | 0 | 2 | 1 | 17 | 18 | 22 | 26 | | 4 | | 1 | | 0 | | | 0 | | 0 | 0 |
|  |  |  |  |  |  |  |  |  |  | |  | |  | |  | | |  | |  |  |
| censors/day | 1 | 2 | 3 | 4 | 5 | 6 | 7 | 8 | 9 | | 10 | | 11 | | 12 | | | 13 | | 14 | 15 |
| N2 | 13 | 0 | 0 | 4 | 0 | 0 | 0 | 0 | 0 | | 0 | | 0 | | 0 | | | 0 | | 0 | 0 |
| d-TMEM CT Tg D | 6 | 0 | 0 | 0 | 0 | 2 | 0 | 0 | 0 | | 0 | | 0 | | 0 | | | 0 | | 0 | 0 |
| d-TMEM CT Tg C | 11 | 0 | 0 | 1 | 4 | 0 | 0 | 0 | 0 | | 0 | | 0 | | 0 | | | 0 | | 0 | 0 |
| TMEM CT Tg B | 2 | 0 | 0 | 1 | 3 | 0 | 1 | 0 | 0 | | 0 | | 0 | | 0 | | | 0 | | 0 | 0 |
| TMEM CT Tg A | 5 | 0 | 0 | 2 | 0 | 0 | 0 | 0 | 0 | | 0 | | 0 | | 0 | | | 0 | | 0 | 0 |
|  |  |  |  |  |  |  |  |  |  | |  | |  | |  | | |  | |  |  |
| Rep 2 (2/8-2/23) |  |  |  |  |  |  |  |  |  | |  | |  | |  | | |  | |  |  |
| Deaths/day | 1 | 2 | 3 | 4 | 5 | 6 | 7 | 8 | 9 | | 10 | | 11 | | 12 | | | 13 | | 14 | 15 |
| N2 | 0 | 0 | 0 | 0 | 0 | 0 | 0 | 1 | 2 | | 9 | | 15 | | 54 | | | 8 | | 5 | 2 |
| TMEM Tg A | 0 | 0 | 0 | 3 | 6 | 29 | 17 | 50 | 11 | | 6 | | 0 | | 0 | | | 0 | | 0 | 0 |
| TMEM Tg B | 0 | 0 | 2 | 8 | 20 | 23 | 6 | 18 | 15 | | 14 | | 7 | | 0 | | | 0 | | 0 | 0 |
| d-TMEM Tg C | 0 | 0 | 0 | 0 | 1 | 3 | 5 | 35 | 34 | | 24 | | 5 | | 0 | | | 0 | | 0 | 0 |
| d-TMEM Tg D | 0 | 0 | 0 | 0 | 5 | 13 | 10 | 32 | 36 | | 19 | | 4 | | 0 | | | 0 | | 0 | 0 |
| dendra Tg | 0 | 0 | 0 | 0 | 0 | 0 | 0 | 0 | 1 | | 2 | | 17 | | 66 | | | 10 | | 2 | 0 |
|  |  |  |  |  |  |  |  |  |  | |  | |  | |  | | |  | |  |  |
| censors/day | 1 | 2 | 3 | 4 | 5 | 6 | 7 | 8 | 9 | | 10 | | 11 | | 12 | | | 13 | | 14 | 15 |
| N2 | 0 | 13 | 2 | 0 | 0 | 1 | 5 | 2 | 0 | | 0 | | 0 | | 0 | | | 0 | | 0 | 0 |
| TMEM Tg A | 0 | 0 | 0 | 0 | 0 | 0 | 0 | 0 | 0 | | 0 | | 0 | | 0 | | | 0 | | 0 | 0 |
| TMEM Tg B | 1 | 5 | 2 | 0 | 0 | 0 | 0 | 0 | 0 | | 0 | | 0 | | 0 | | | 0 | | 0 | 0 |
| d-TMEM Tg C | 0 | 1 | 1 | 1 | 1 | 0 | 0 | 1 | 0 | | 0 | | 0 | | 0 | | | 0 | | 0 | 0 |
| d-TMEM Tg D | 0 | 1 | 1 | 0 | 0 | 0 | 1 | 0 | 0 | | 0 | | 0 | | 0 | | | 0 | | 0 | 0 |
| dendra Tg | 0 | 0 | 0 | 0 | 0 | 0 | 0 | 0 | 0 | | 1 | | 0 | | 0 | | | 0 | | 0 | 0 |

|  |  |  |  |  |  |  |  |  |  |  |  |  |  |  |  |
| --- | --- | --- | --- | --- | --- | --- | --- | --- | --- | --- | --- | --- | --- | --- | --- |
| Rep 3 (3/14-3/29) |  |  |  |  |  |  |  |  |  |  |  |  |  |  |  |
| Deaths/day | 1 | 2 | 3 | 4 | 5 | 6 | 7 | 8 | 9 | 10 | 11 | 12 | 13 | 14 | 15 |
| N2 | 0 | 0 | 0 | 0 | 0 | 0 | 0 | 1 | 2 | 6 | 14 | 25 | 43 | 20 | 4 |
| TMEM Tg A | 0 | 0 | 0 | 5 | 18 | 24 | 18 | 23 | 14 | 9 | 10 | 1 | 0 | 0 | 0 |
| TMEM Tg B | 0 | 0 | 1 | 3 | 36 | 22 | 17 | 10 | 10 | 4 | 9 | 0 | 0 | 0 | 0 |
| d-TMEM Tg C | 0 | 0 | 1 | 9 | 10 | 16 | 11 | 23 | 16 | 7 | 7 | 0 | 0 | 0 | 0 |
| d-TMEM Tg D | 0 | 0 | 0 | 8 | 7 | 7 | 14 | 23 | 29 | 14 | 7 | 0 | 0 | 0 | 0 |
| dendra Tg | 0 | 0 | 0 | 0 | 0 | 0 | 1 | 2 | 2 | 3 | 24 | 51 | 23 | 8 | 0 |
|  |  |  |  |  |  |  |  |  |  |  |  |  |  |  |  |
| censors/day | 1 | 2 | 3 | 4 | 5 | 6 | 7 | 8 | 9 | 10 | 11 | 12 | 13 | 14 | 15 |
| N2 | 0 | 0 | 0 | 2 | 0 | 4 | 0 | 0 | 0 | 0 | 0 | 0 | 0 | 0 | 0 |
| TMEM Tg A | 3 | 0 | 0 | 0 | 0 | 0 | 0 | 0 | 0 | 0 | 0 | 0 | 0 | 0 | 0 |
| TMEM Tg B | 8 | 0 | 0 | 1 | 0 | 1 | 0 | 0 | 0 | 0 | 0 | 0 | 0 | 0 | 0 |
| d-TMEM Tg C | 2 | 0 | 7 | 1 | 1 | 0 | 0 | 0 | 0 | 0 | 0 | 0 | 0 | 0 | 0 |
| d-TMEM Tg D | 5 | 0 | 4 | 1 | 0 | 0 | 0 | 0 | 0 | 0 | 0 | 0 | 0 | 0 | 0 |
| dendra Tg | 0 | 0 | 2 | 0 | 0 | 0 | 0 | 2 | 0 | 0 | 0 | 1 | 0 | 0 | 0 |

**Supplemental Table S2: Deaths and Censures per Day For *C. elegans* Strains During Lifespan Assay.** Worms counted as dead when one did not respond to gentle touch from platinum wire. Worms that burst from FUDR treatment or worms that crawled off of the plate during assay were censored.
